# Metal Nanoparticles for Electrochemical Sensing: Progress and Challenges in the Clinical Transition of Point-of-Care Testing

**DOI:** 10.3390/molecules25245787

**Published:** 2020-12-08

**Authors:** Tamanna Islam, Md. Mahedi Hasan, Abdul Awal, Md Nurunnabi, A. J. Saleh Ahammad

**Affiliations:** 1 Department of Chemistry, Jagannath University, Dhaka 1100, Bangladesh; islamtamanna1992@gmail.com (T.I.); mdhasanmahedi83@gmail.com (M.M.H.); sayem2021@gmail.com (A.A.); 2 Department of Pharmaceutical Sciences, School of Pharmacy, University of Texas at El Paso, El Paso, TX 79902, USA; 3 Department of Biomedical Engineering, University of Texas at El Paso, El Paso, TX 79968, USA; 4 Department of Environmental Science & Engineering, University of Texas at El Paso, El Paso, TX 79968, USA

**Keywords:** electrochemical biosensors, point-of-care testing, metal nanoparticles, cancer biomarkers, glucose, novel coronavirus

## Abstract

With the rise in public health awareness, research on point-of-care testing (POCT) has significantly advanced. Electrochemical biosensors (ECBs) are one of the most promising candidates for the future of POCT due to their quick and accurate response, ease of operation, and cost effectiveness. This review focuses on the use of metal nanoparticles (MNPs) for fabricating ECBs that has a potential to be used for POCT. The field has expanded remarkably from its initial enzymatic and immunosensor-based setups. This review provides a concise categorization of the ECBs to allow for a better understanding of the development process. The influence of structural aspects of MNPs in biocompatibility and effective sensor design has been explored. The advances in MNP-based ECBs for the detection of some of the most prominent cancer biomarkers (carcinoembryonic antigen (CEA), cancer antigen 125 (CA125), Herceptin-2 (HER2), etc.) and small biomolecules (glucose, dopamine, hydrogen peroxide, etc.) have been discussed in detail. Additionally, the novel coronavirus (2019-nCoV) ECBs have been briefly discussed. Beyond that, the limitations and challenges that ECBs face in clinical applications are examined and possible pathways for overcoming these limitations are discussed.

## 1. Introduction

Biosensors are chemical sensors that utilize biomolecules as the target recognizing component and a transducer that produce an identifiable signal through their interaction [1,2]. In the case of electrochemical biosensors (ECBs), the transducer converts the chemical signal to an electrical signal that allows for qualitative and quantitative identification of the target biomolecules [1,3,4,5]. With the increasing risk of cancer, diabetes, viral infections, and other pathogenic diseases, point-of-care testing (POCT) systems have become essential in the health sector [4,6,7,8]. As a result, research in ECBs has seen an exponential growth because they are inexpensive, provide fast and accurate responses, require almost no sample preparation, and are easy to use [3,8,9,10,11,12,13].

**Figure 1:**
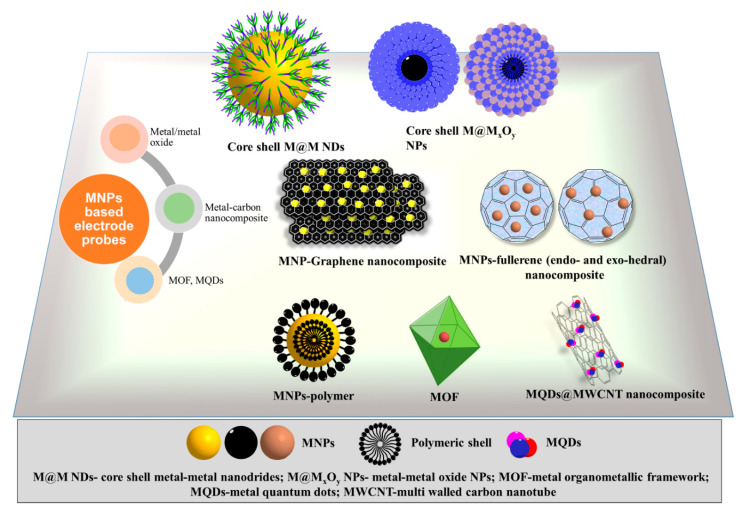
Different metal nanoparticle (MNP)-based composite materials as electrode probes for electrochemical biosensors (ECBs).

ECBs often take advantage of the unique chemical properties possessed by nanomaterials [14,15,16]. Particularly, metal nanoparticles (MNPs) are mostly used due to their biocompatibility, low toxicity, excellent conductivity, and high surface area [17,18,19,20,21]. Among many, gold (AuNPs), silver (AgNPs), and platinum NPs (PtNPs) are some of the most commonly utilized in ECBs [21,22,23,24,25,26,27,28]. In fabricating biosensors, these MNPs often provide the anchoring site for biorecognition components such as antibodies, enzymes, single stranded RNA (ssRNA) and DNA (ssDNA), aptamers, and affibodies [21,22,23,24,27,29,30,31]. The effectiveness and stability of these biochemical interactions are largely dependent on the physicochemical properties of the MNPs [1,20,32]. This is why material researchers have devised unique strategies for controlling size, shape, and other structural features of these MNPs [32,33]. However, MNPs are often combined with a scaffold for increasing stability and catalytic activity that is usually made of nanostructured material [34,35,36,37]. Of these, the carbon nanostructures are the most popular candidate due to their availability, good conductivity, and stability [9,35,38,39,40]. The various carbon nanostructures used are 0D fullerenes, 1D carbon nanotubes (CNTs), 2D graphene (GR), and 3D graphite materials [41,42,43,44]. In ECBs, the composites of MNPs and these carbon nanostructures are used to anchor the biorecognition components to the transducer that converts the chemical signal to electronic signal. Figure 1 depicts the various MNPs and their composites that are often utilized in ECBs.

ECBs can be manufactured in miniaturized size that can be used as POCT devices for clinical purposes [45,46]. These POCT devices are often fabricated on paper strips or carbon paste electrodes based on lab-on-chip principles and can be used with a portable electroanalytical device [46,47]. Electrochemical glucose biosensors are one of the most successful and promising examples of this technology [48]. However, the practical applications of ECBs for a wide variety of bioanalytes have not been completely realized. Besides the cost of the electrodes, the key technical factors that determine the applicability of ECBs in clinical purposes are: ease of preparation, sensitivity, accuracy, reproducibility, and stability of the modified electrode [49,50,51]. However, the use of biomolecules makes it very challenging to meet all these criteria.

The review is devoted to discussing how using MNPs and carbon composites can help to overcome the limitations of ECBs. The field of ECBs is enormous; hence a general classification of the ECBs is considered for simplification purposes. The progress in the ECB research has also been discussed to provide the reader with a better understanding of the development process over the last decade. The design strategies for tailoring the properties of MNPs and carbon nanostructures that influence the sensing ability has also been explored. The review also discusses the advances in ECBs for sensing small biomolecules and cancer biomarkers. Beyond these, the challenges and perspective course of actions to overcome them have been explored. Hence, the authors hope that the discussions and concepts presented in this review would envisage the fabrication of ECBs that can be applied for POCT.

## 2. Electrochemical Biosensors

**Figure 2:**
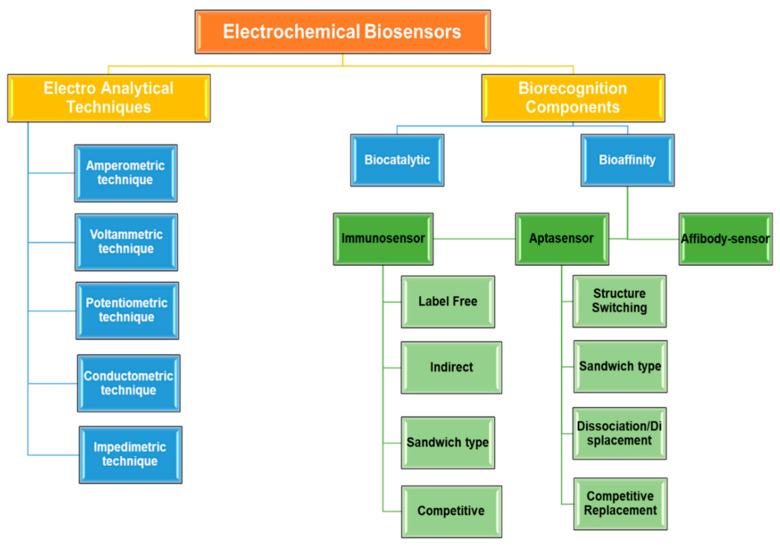
Classification ECBs. Left-hand side shows the classification with respect to electroanalytical techniques (EATs) and the right-hand side shows classification with respect to biorecognition components (BRCs).

ECBs can achieve high selectivity and accuracy through combining bio-selectivity of biomolecules and sensitivity of the electroanalytical techniques (EATs) [9,52]. Application of ECBs encompasses a wide variety of research area from small biomolecules (dopamine, glucose, xanthine, etc.) to cancer biomarkers and other large biological systems [3,25,26,27,28,29,30,31,32,33,34,35,36,37,42,44]. These biosensors vary from each other based on the use of biorecognition components (BRC) and EAT for the detection process [1,4]. Typically, ECBs comprise three electrodes (working electrode (WE), reference electrode (RE), and counter electrode (CE)) that are placed in contact with the analyte solution in an electrochemical cell [1,6,53]. These three electrodes are connected with an electrochemical workstation that is capable of applying potential and measuring the electrochemical changes due to electron transfer at the interfacial region between the WE (transducer) and solution [46,51]. The circuitry component of the workstation converts such chemical changes at the WE into readable data in terms of current, potential, or conductivity with respect to the WE [51]. That is why understanding the properties of the WE is most important when discussing about ECBs. The WE acts as the transducer which can convert electrochemical reactions into electrical signals [53,54]. The WE is modified with various BRCs and utilizes different EATs to make them sensitive and selective towards a particular type of analyte. In this review, ECBs have been categorized to simplify these wide varieties. Initially, ECBs can be broadly categorized into two classes, based on the type of EATs and BRCs employed. The classification of ECBs based on EATs and BRCs used are represented by a hierarchical list in Figure 2.

### 2.1. Classification of ECBs Based on BRCs

When considering how the biorecognition systems work, the ECBs can be categorized into two classes:

1. ECBs modified with biocatalysts
2. ECBs operating through bioaffinity

#### 2.1.1. ECBs Modified with Biocatalysts

**Figure 3:**
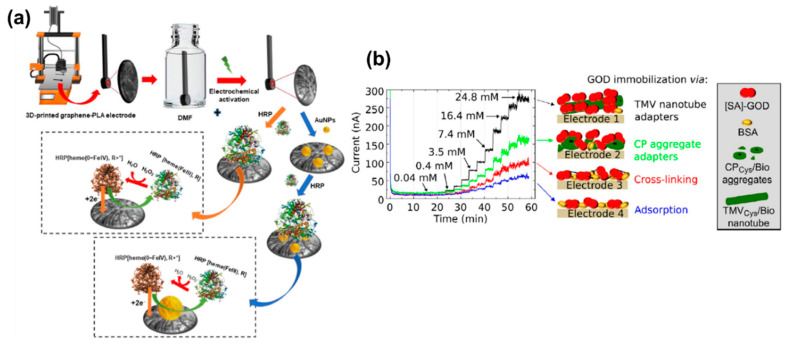
ECBs for H_2_O_2_ and glucose detection. (**a**) Fabrication of the screen printed H_2_O_2_ sensor and H_2_O_2_ detection mechanism through interaction with the horseradish peroxidase (HRP) enzyme [62]. (**b**) Amperometric response for different modifications of the electrochemical glucose biosensors [63]. Reprinted with permission from [62,63], Copyright © 2020 and 2019 Published by Elsevier B.V. DMF: Dimethylformamide, GOD: Glucose oxidase, CP: Coat proteins, TMV: Tobacco mosaic virus, BSA: Bovine serum albumin.

Biocatalysts are BRCs that can produce electroactive species by interacting with biomolecules [55,56]. Enzymes, cells, tissues, and small biomolecules are often used as biocatalysts in the ECBs [55,56]. These ECBs most commonly employ impedimetric, voltammetric, and amperometric methods in their analyses [56,57]. Preparing ECBs with biocatalysts is often cost-effective, simple, and easily scalable to make POCT devices [58]. Of the various biocatalysts used, enzymes are most popular [59]. This is because of enzymes are amino acids that are capable of inducing biochemical catalysis [1,56]. They are capable of interacting with electrochemically inactive bioanalytes and producing electroactive species [26,27,60]. Enzymes often significantly increase the rate of reaction and the kinetic parameters can be readily investigated with simple EATs [1,60]. However, these enzymes are often very sensitive to temperature, pH of the solution, and humidity [61]. That is why it is important to maintain optimal conditions while preparing and using this type of sensors. Researchers are working towards fabricating enzymatic sensors that are more tolerant to these limiting factors. Marzo and coworkers have reported the fabrication of horseradish peroxidase (HRP)-based ECB for highly sensitive H_2_O_2_ detection [62]. The sensor uses a composite of AuNP-HRP that is immobilized on 3D screen printed (3D-SP) GR-polylactic (PLA) substrate. The HRP acts as the catalytic enzyme that induces electron transfer from H_2_O_2_. The 3D-SP electrode fabrication and its H_2_O_2_ detection mechanism are illustrated in Figure 3a. The 3D-SP sensor showed relatively good stability [62]. An enzymatic ECB has been reported, where virus (tobacco mosaic virus (TMV)) was used as a carrier to enhance the sensitivity and stability of glucose oxidase (GOx) enzyme for glucose detection [63]. The TMV carrier containing a glucose sensor showed almost double the current response for the same concentration of glucose compared to the non-TMV containing sensor [63]. Figure 3b shows the glucose sensor chips and their response towards glucose detection. Further research to the advancement of biocatalytic sensors have made them a promising candidate for POCT.

#### 2.1.2. ECBs Operating through Bioaffinity

In these biosensors, a biorecognition element is used that can strongly bind with the target biomolecules to produce a detectable electrical signal [64]. Affinity ECBs utilize antigens to bind with antibodies, various oligomers, ssRNA or ssDNA, and membrane receptors to bind specifically [1,4,65]. These bioaffinity sensors are often used when biocatalytic systems are not applicable. For instance, there are many biomolecules for which there is no enzyme that can selectively catalyze them [1,65,66]. Based on the type of biorecognition molecules used, the bioaffinity-based ECBs can be classified as the following:

- Immunosensors
- Aptasensors
- Affibody-based sensors

**Figure 4:**
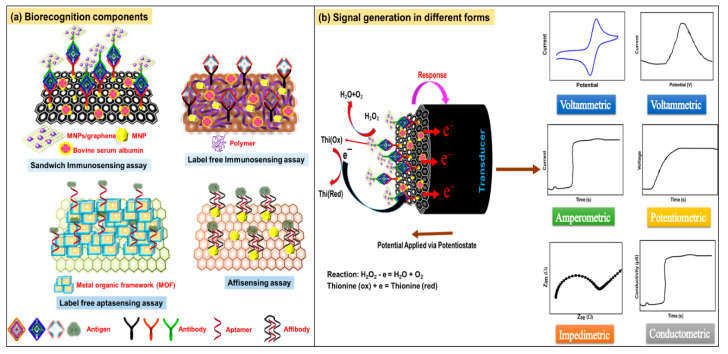
Simplified illustration of different biorecognition components designed based on sandwich, label-free immunosensing assay, aptasensing assay and affisensing assay (**a**) and the process of signal generation and different electroanalytical techniques used for ECBs (**b**).

Figure 4a shows schematic diagrams of the marker (signal inducer)-labeled sandwich and label-free immunosensor, aptasensor and affisensor.

**Figure 5:**
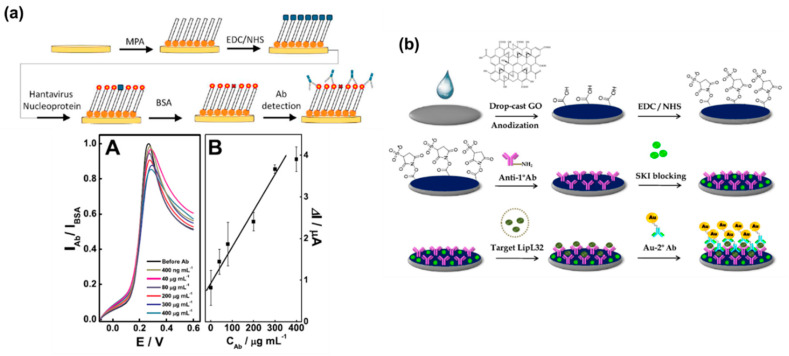
Label-free and sandwich type immunosensor fabrication process. (**a**) Label-free immunosensor preparation through antibody immobilization for the detection of hantavirus [73]. (**b**) Schematic representation of the sandwich type electrochemical immunosensor fabrication process for the sensitive detection of LipL32 which is responsible for leptospirosis [80]. Reprinted with permission from [73,80], Copyright © 2020 Published by Elsevier B.V. MPA: 3-mercaptopropionic acid, EDC: N-(3, Dimethylaminopropyl)-N-ethyl-carbodiimidehydrochloride, NHS: N-hydroxysuccinimide ester, Ab: Antibody, GO: Graphene oxide.

Immunosensors utilize antibody-antigen (Anb–Ang) interactions for producing detectable electrochemical signals [67]. Immunosensors take advantage of the strong selective chemical affinity between antigen and antibody [68]. As a result, immunosensors are highly selective, very sensitive, and accurate in their detection. Immunosensors can be categorized as traditional label-free/direct, indirect, sandwich, and competitive type based on the mode of operation [69]. Again, based on the change of signal response immunosensors can be categorized as “signal on” and “signal off” [68,69]. In the case of label-free immunosensors, the electrode surface is tailored with an antibody that can bind with the specific antigen [70]. The electrolyte solution usually contains an electroactive redox pair that is responsible for the electrochemical signal. The redox pair interaction with the electrode surface changes based on the concentration of the antigen present on the solution. As a result, the electronic signal varies and allows for quantitative detection of the antigen [71]. The setup can be reversed, where the antigen is immobilized for specific antibody detection. In the direct label-free method, the antigen is immobilized on the substrate and allowed to interact with the antibody [72]. An antibody of hantavirus has been detected from serum solution using an AuNP-hantavirus antigen-modified electrode [73. The sensor used a linear sweep voltammetry (LSV) technique and showed linearity for hantavirus antibody detection for 0.4 to 300 µg/mL. Sensor fabrication and hantavirus detection is shown in Figure 5a. The reported sensor also showed good stability over 21 days [73]. In another work, prostate specific antigen (PSA) detection was carried out with direct label-free method by Camilo et al [72]. The report showed that AuNPs and layer by layer (LBL)-assembled nanostructures can be used for signal amplification in a direct immunosensor detection system while simultaneously lowering the number of biomolecules (antibody) needed. Their proposed sensor required approximately 10 times fewer antibodies compared to traditional PSA immunosensors [72]. Label-free immunosensors are also employed for detecting proteins, hormones, bacteria, etc. [74].

In the case of indirect immunoassays, the quantitative analysis of antigens is carried out by measuring the changes in electrochemical signal due to the conjugation of a labeled secondary antibody with the primary antibody which is already bound to antigen [75]. The design of indirect immunoassay follows two-step binding strategies in which the primary label-free antibody binds to antigen which is first immobilized on the substrate. Later, a labeled secondary antibody is immobilized on it which can recognize the primary antibody and subsequently bind to it. The secondary antibody can be labeled with various electroactive compounds or enzymes which helps in generating the desired signal.

A competitive immunosensing process utilizes the ability of antigen–antibody binding affinity along with the catalytic properties of biocatalysts [76]. Typically, different labeled secondary antibodies compete to bind with inadequately available primary antibody sites. AuNP-modified electrodes have been utilized for the detection of phenolic estrogens through indirect competitive binding processes [75]. The four phenolic estrogens conjugated with the secondary antibody and the binding affinity followed: diethylstilbestrol > dienestrol > bisphenol A > hexestrol. This work utilized a differential pulse voltammetry (DPV) technique [75]. In another work, Hou et al. reported a direct competitive ECB that utilized an electrochemical impedance spectroscopy (EIS) technique for detecting chlorpyrifos [77]. Chlorpyrifos antibodies were initially anchored on a glassy carbon electrode (GCE) surface. Analyte competitor was prepared by combining spherical AuNPs with HRP, bovine serum albumin (BSA), and chlorpyrifos. This analyte competitor then competed with chlorpyrifos to bind with the anchored antibody. This resulted in the formation of insoluble 4-chloro-1(4H)-naphthalenone through biocatalytic process in the presence of H_2_O_2_ and 4-chloro-1-naphthol. The proposed electrode linear range from 1.0 × 10^−3^ ng mL^−1^ ~10 ng mL^−1^ [77]. The competitive immunoassay method can be utilized with other immunoassay method for amplifying the signal. This is very effective in lowering the limit of detection (LOD) of ECBs.

Although label-free immunosensors are very selective, they are not adequately sensitive [78]. Hence, sandwich type immunosensors were conceived to overcome this limitation [1,78]. Similar to the label-free system, the antibody (Anb1) is first immobilized on the ECB surface and allowed to interact with the antigen (Ang). However, a second antibody (Anb2) is introduced to the system that interacts only with the Anb1–Ang sites on the electrode surface to produce the sandwich (Anb1–Ang–Anb2) [68,79]. As a result, the change in electronic signal is amplified and the sensitivity is improved. Jampasa et al. developed an ECB for the sensitive detection of LipL32 protein through a “signal on” process [80]. The sensor utilized a graphene oxide (GO) layer for immobilizing the Anb1. The modified electrode was allowed to interact with the antigen. Finally, Au conjugated Anb2 was introduced to the electrode system. The electrode fabrication process is shown in detail in Figure 5b. This interaction process ensured selectivity and high sensitivity. The DPV technique was utilized for the detection process. The sensor showed a stable current response towards LipL32 for over 14 days [80].

Aptasensors were developed to overcome the limitations posed by the immunosensors [81,82]. POCT devices need to be cheap, robust, and easily scalable [65,83]. Using immunosensors it is often difficult to fulfill these criteria. Aptasensors use aptamers (ssRNA, ssDNA, and peptides containing 15–40 bases) with unique binding sites that utilize their nucleic acid arrangements for interacting selectively with target biomolecules [1,84]. Aptamer spatial configurations are changed to enable the best interaction with the target biomolecules [84]. Additionally, aptamers are more stable than antigens, and can easily recover their active spatial configuration after usage thereby allowing for the reuse of the same electrode multiple times [85]. Based on how the immobilized aptamers interact with the target analyte, Han and coworkers proposed the following categories: (a) spatial configuration rearrangement of aptamers based on target interaction [86,87,88]; (b) sandwich type interactions [89,90,91]; (c) dissociation or displacement of aptamers through target interaction [92,93]; and (d) competitive replacement of aptamers [14,94,95,96].

**Figure 6:**
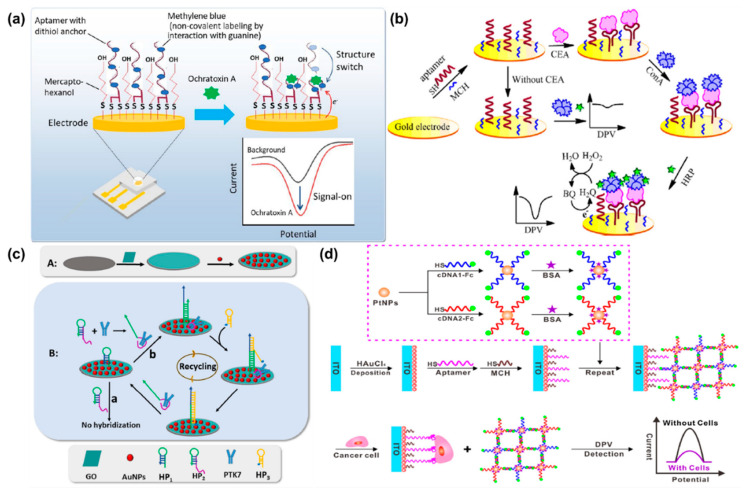
Electrochemical aptasensors for the detection of biomolecules. (**a**) Aptamer anchored on gold electrode surface for Ochratoxin A (OHA) detection through structure switching of the aptamer [88]. (**b**) ECB for the sensitive detection of carcinoembryonic antigen (CEA) through label-free sandwich method [89]. (**c**) Aptamer displacement strategy-based sensor for the detection of PTK-7 [93]. (**d**) Schematics of a label-free competitive aptamer cytosensor design and detection process of hepatocellular carcinoma (HepG2) cells [96]. Reprinted with permission from [88], Copyright © 2020 Published by J-STAGE, [89], Copyright © 2020 Published by Elsevier B.V., [93], Copyright © 2020 Published by Springer Nature, [96], Copyright © 2020 Published by ECS. MCH: 6-mercapto-1-hexanol, PTK-7: protein tyrosine kinase-7, HP: hairpin probe.

In the case of type-a biosensors, the immobilized aptamers change their configuration with respect to interactions with the target biomolecules [87]. Mazaafrianto and co-workers developed an aptasensor for detecting ochratoxin A (OHA) based on structure switching [88]. The proposed sensor was able to obtain an LOD of 113 pm through the “signal on” method. Figure 6a shows the OHA sensor setup and its interaction process for electrochemical signaling. OHA interaction induced the structure change in the aptamer that then allowed interaction with methylene blue (MB) that resulted in the increased signal [88].

Similar to immunosensor, sandwich type aptasensors (type b) also utilize signal amplification through a double interaction system [69]. Research strategies have been focused on developing label-free sandwich aptasensors for cost-effective and rapid biomolecule detection. Wang et al. proposed an antibody and label-free sandwich sensor for the detection of carcinoembryonic antigen (CEA) cancer biomarker [89]. In this setup, the aptamer was deposited on the Au electrode surface and allowed to interact with CEA. The sensor showed an increased DPV response when *Concanavalin A* (conA) was allowed to interact with the Au/aptamer–CEA conjugate. However, in the absence of CEA, conA did not show any interaction with the Au/aptamer electrode system shown in Figure 6b. The sensor showed very good selectivity along with a low LOD of 3.4 ng/mL [89].

For type-c ECB systems, the aptamer probe is modified through displacement/dissociation at certain sequences in the presence of the target biomolecules [96]. This allows for high selectivity towards the analyte and amplification of the electrochemical signal. In a work by Li et al. the sgc8 aptamer was used for modifying a hairpin probe (HP2) and detecting protein tyrosine kinase-7 (PTK-7) [93]. HP2 was immobilized on the GCE surface along with HP1. In the presence of PTK-7, the HP2 may undergo structural change exposing the aptamer that hybridized with the HP1. Finally, a redox probe carrying HP3 is introduced that upon interaction produced suitable voltammetric signals. Besides the very low LOD of 1.8 fM, the sensor surface is regenerated through the removal of PTK-7 at the end of each cycle [93]. The ECB fabrication process and PTK-7 detection mechanism are shown in Figure 6c.

For type-d ECBs, the target analyte replaces the aptamer to produce the desired electrical signal [94]. Figure 6d shows the mechanism for such a sensor that was used for detecting hepatocellular carcinoma (HepG2) tumor cells through the “signal off” process [96]. An LBL assembly system was used where AuNPs were initially deposited on ITO (indium tin oxide) electrodes along with a TLS11a aptamer. This was then allowed to interact with the LBL assembly of PtNP-Fc-labeled cDNA (complementary DNA). When no tumor cells were present, the PtNP assembly gave a high current response. However, in the presence of tumor cells the cDNA could no longer effectively bind with the aptamer due to denaturation of the double strand DNA. This resulted in a decreased current signal that was linearly proportional to the logarithm of the HepG2 cell concentration. [96].

**Figure 7:**
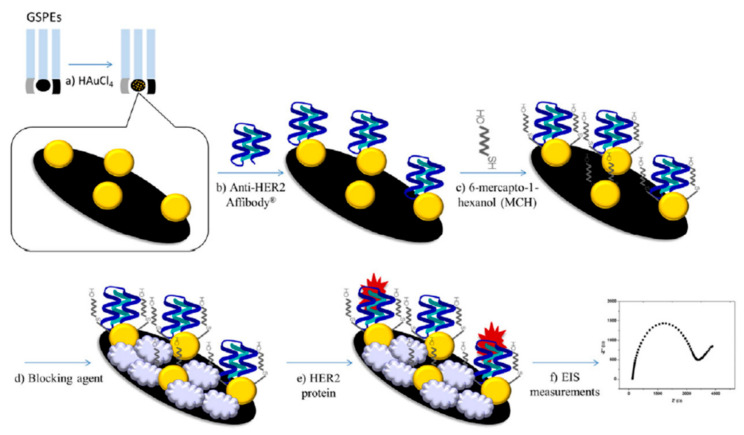
Affibody-sensor for the detection of human epidermal growth factor receptor 2 (HER2) biomarker. (**a**) Preparation of AuNP–graphite strip through electrodeposition. (**b**) Anti-HER2 immobilization over the AuNP–graphite strip. (**c**) Formation of MCH self-assembled monolayer with the anti-HER2 AuNP–graphite strip. (**d**) Addition of blocking agent to the electrode strip. (**e**) Interaction with HER2 and (**f**) the corresponding impedance signal [100]. Reprinted with permission from [100], Copyright © 2020 Published by Elsevier B.V. GSPEs: Graphite screen-printed electrodes, EIS: Electrochemical impedance spectroscopy.

Affibody-based sensors are a result of using antibody mimicking bioengineered small protein (6 to 7 kDa) molecules to overcome the limitations of immunosensors [97]. These affibodies are engineered according to the need and have high binding affinity, selectivity, and survivability in high temperature conditions [98]. Antibodies typically contain disulfide bonds that lead to poor heat stability [1]. However, only a small portion of the multidomain protein structure of antibodies is used in antigen detection [1,98]. This is where affibody technology comes into use. The parts of antibodies that are responsible for their affinity and selectivity towards antigens are engineered in vitro. These affibodies are often paired with various metal nanoparticles to further enhance their efficacy [99]. An impedimetric strip ECB for human epidermal growth factor receptor 2 (HER2) biomarker that utilized affibody as the biorecognition element is shown in Figure 7 [100]. AuNPs were used for immobilizing the anti-HER2 affibodies. This resulted in selective interaction with the HER2. Because of that, the impedimetric charge transfer resistance increased linearly with increasing HER2 concentration. Analysis of the experimental results provided an LOD of 6 µg/L for the proposed sensor. Compared to conventional immunosensors, the affibody sensor was more sensitive, provided a more rapid response, and higher specificity [100].

### 2.2. Classification of ECBs Based on EATs

A wide variety of EATs are currently employed in ECBs [53]. These techniques are sometimes combined to work synergistically to further amplify the electronic signals [53,54]. Therefore, based on the different EATs utilized, ECBs can be categorized as follows:

- Amperometric technique
- Voltammetric technique
- Potentiometric technique
- Conductometric technique
- Impedimetric technique

Figure 4b shows a schematic representation of ECB sensing process and the different EATs used in them. Below, these techniques are discussed in detail.

#### 2.2.1. Amperometric Technique

**Figure 8:**
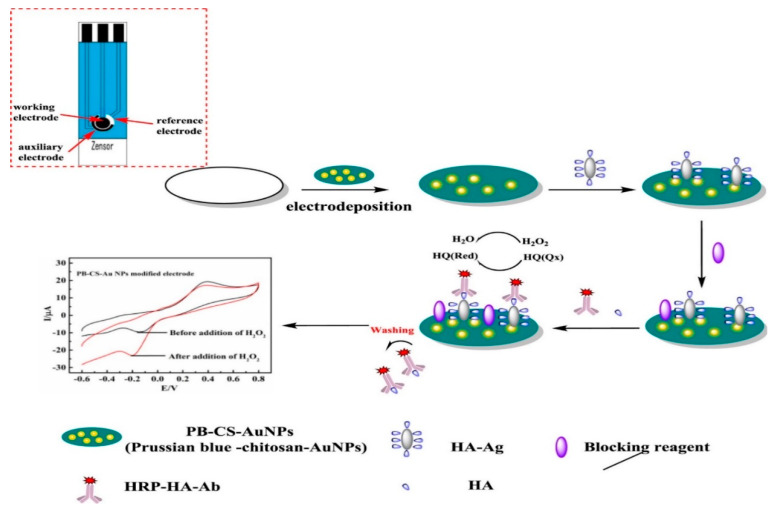
Fabrication of ECB strip for histamine (HA) detection. Portable immunosensor developed for the (point-of-care testing) POCT of HA with PB-CS-AuNPs [105]. Reprinted with permission from [105], Copyright © 2020 Elsevier Ltd. B.V.

This is a sensitive EAT that utilizes an applied potential for inducing oxidation or reduction of the target analyte and the response is observed as a change of current signal with respect to time and the analyte concentration [101]. It is one of the most popular EATs because it offers sensitive detection and is very simple to use [101,102]. The LOD of the amperometric technique is in the range of 10^−5^ M [103]. The use of specific analyte is advantageous because it allows for limiting interference [104]. At the same time, the charging current is also minimized within a few hundred seconds allowing for a very low limit of detection (LOD) [1,102]. Because of these advantageous properties, ECBs have often utilized amperometric techniques. For instance, a portable ECB was proposed by Dong and coworkers for the sensitive and selective detection of histamine (HA) through an AuNP–chitosan–Prussian blue-modified electrode system [105]. The electrode used an HA antigen that competitively interacted with an HRP-labelled HA antibody for HA detection using an amperometry technique within the 0.01 to 100 µg/L linear range [105]. Figure 8 shows a schematic presentation of the portable sensor fabrication process and its application for HA detection. Combined hydrodynamic and amperometry techniques can further enhance the sensitivity and lower the LOD [106]. A hydrodynamic amperometry technique-based aqueous uranyl ion ECB was reported, which showed a higher sensitivity and lower detection limit compared to steady state amperometric systems that were previously reported [106].

#### 2.2.2. Voltammetric Technique

**Figure 9:**
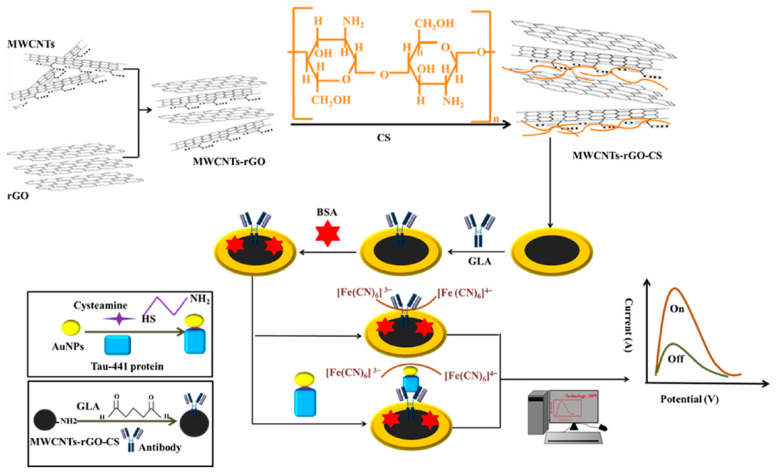
A “signal off” ECB for the voltammetric detection of Tau-441 protein. Assembly of multi-walled carbon nanotubes-reduced graphene oxide-chitosan-antibody (MWCNT–rGO–CS) over the gold electrode for the voltammetric detection of AuNP–Tau-441 conjugate [112]. Reprinted with permission from [112], Copyright © 2020, Springer Nature.

These are EATs where a certain potential region is scanned, and the signal is displayed in the form of a peak or a plateau [107]. The current response is proportional to the concentration of the analyte present in the system [13,107]. The most commonly used voltammetric techniques include linear sweep voltammetry (LSV), cyclic voltammetry (CV), DPV, and square wave voltammetry (SWV) [108,109,110]. Although the principle is same for all the techniques, they differ in the way that the potential region is scanned. Based on the scanning method, the most sensitive are DPV and SWV [111]. The detection limits for LSV, CV, DPV, and SWV are 10^−5^, 10^−5^, 10^−7^, and 10^−8^ M, respectively [103]. DPV-based highly sensitive ECB was reported for the detection of Tau-441 protein, which is correlated to cognitive disorder [112]. The proposed ECB utilized an Au electrode that was modified with multi-walled carbon nanotubes-reduced graphene oxide (MWCNT–rGO) and Tau-441 specific antibody. The sensor showed a linear range from 0.5–80 fM with an LOD of 0.46 fM [112]. The voltammetric electrode preparation process is shown in Figure 9. Such low-level detection of bioanalytes with voltametric-based ECBs shows their potential for application in POCT.

#### 2.2.3. Potentiometric Technique

**Figure 10:**
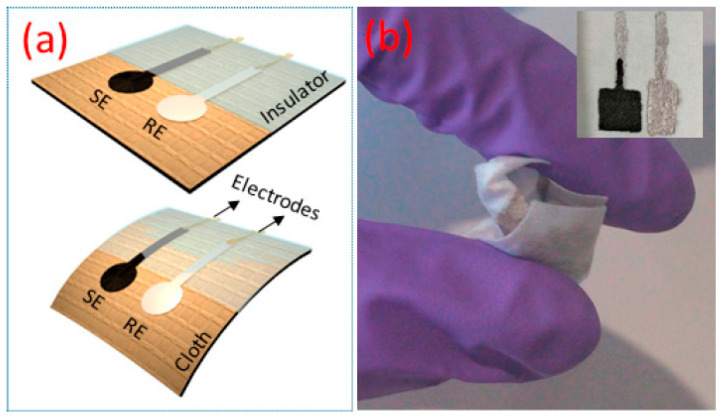
Schematic representation and photograph of the wearable ion selective electrodes (ISE) pH sensor. (**a**) Schematic of the pH sensor where Ag was deposited on the cloth. The sensing electrode (SE) and reference electrodes (RE) are screen printed over the Ag substrate. (**b**) Picture of the flexible cloth sensor. Inset shows the SE and RE electrodes [117]. Reprinted with permission from [117], Copyright © 2020, MDPI.

For the potentiometric technique, the change of potential in the electrochemical cell is measured, while the current change is minimal [113]. The potentiometric sensors are also known as ion selective electrodes (ISEs) because they are often designed to generate responses with respect to the change in concentration of a specific ion [113,114]. Their setups are different from the traditional amperometric and voltammetric cells, because they often utilize two reference electrodes that measure the potential change with respect to the target analyte concentration in the cell [113,115]. These ISEs can be converted to ECBs by modifying the electrode with biocatalysts that interact with biomolecules to produce ions that the ISEs can detect [1,113]. Like other ECBs, they can also operate independent of sample volume, have a low LOD, small size, and produce a rapid response. On top of these, potentiometric ISEs are able to provide information regarding the concentration of free ions or ion activity in the cell [114,116]. Manjakkal et al. reported the fabrication of a potentiometric pH sensor that can be used as a wearable device [117]. The sensor showed excellent stability to washing and a good sensitivity of 4 mV/pH in the pH range of 6–9 [117], making it an excellent candidate for POCT for various bioanalytes through the incorporation of proper biorecognition component. The fabricated wearable device is shown in Figure 10.

#### 2.2.4. Conductometric Technique

**Figure 11:**
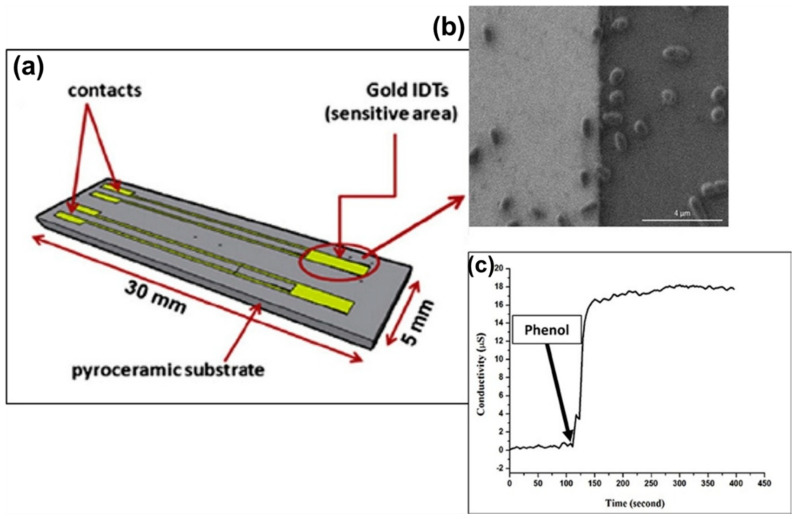
Conductometric ECB for the detection of phenol. (**a**) The strip gold electrode with 20 nm thick intermediate titanium (IDT) for phenol detection. (**b**) SEM image of bacteria over the glassy carbon electrode (GCE) (grey) and gold (dark) conductometric sensor. (**c**) The change in conductance with respect to the addition of phenol [121]. Reprinted with permission from [121], Copyright © 2020 Elsevier Ltd.

The change of conductance in the electrochemical setup is measured as a response of interactions between the BRC and analyte [118]. These types of ECBs usually use catalytic biorecognition modifications that result in the change of ionic strength in the cell [118,119]. This change is then measured to determine the biomolecules qualitatively and quantitatively [73,120]. Kolahchi et al. have developed a miniaturized conductometric electrodes for phenol detection [121]. The optical microscopic image and electrochemical setup of the device is shown in Figure 11. In this setup, they used AuNP-immobilized bacteria (*Pseudomonas* sp. (GSN23)) as the biorecognition component for the detection process. This setup enabled bypassing of the micro-extraction process required for phenol detection with a simple dilution procedure [121]. Conductometric sensors have also been used for the detection of biomolecules from human serum and urine samples, and pathogens from foods for biosecurity purposes [118,122,123].

#### 2.2.5. Impedimetric Technique

**Figure 12:**
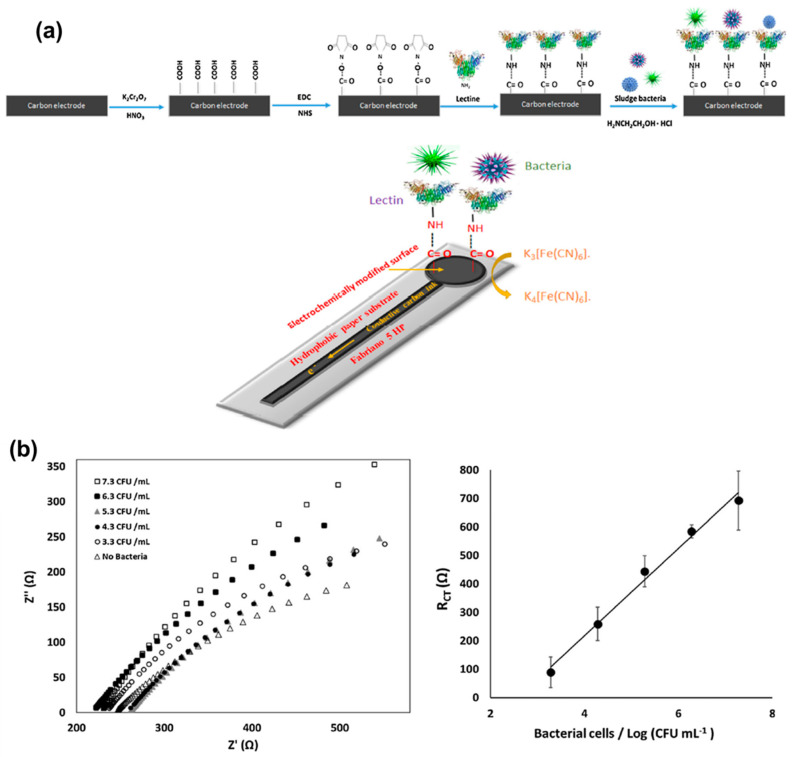
Paper-based impedimetric ECB for the detection of bacterial colonies in the water. (**a**) Schematic representation of the fabrication of paper-based ECB. (**b**) Change of impedance signal with the bacterial colony concentration variation and the corresponding charge transfer resistance (R_CT_) vs concentration calibration plot [126]. Reprinted with permission from [126], Copyright © 2020 Published by Elsevier B.V.

In the impedimetric technique, the changes in resistance and capacitance at the interfacial region of the working electrode are measured with respect to analyte concentration [68]. This is also known as electrochemical impedance spectroscopy (EIS) technique. The measurements are carried out through the application of an alternating current as the exciting factor that perturbs the voltage as a function of frequency (1 × 10^−5^ to 0.7 V) [1,124]. This is one of the most popular techniques used in bioaffinity sensors (immunosensor and aptasensors), because of its high sensitivity to slight changes in impedance [125]. A paper-based impedimetric ECB was developed for the rapid and on-site detection of bacterial contaminations in drinking water [126]. The paper strip was initially carboxylated, and was then used for immobilizing *Concanavalin A* (conA) lectin. The paper sensor setup process is shown in Figure 12. The impedimetric sensor showed variation of resistance over a wide concentration window with a low LOD of 1.9 × 103 CFU/mL [126]. Impedimetric ECBs are frequently employed for detecting small biomolecules and various biomarkers [127].

## 3. MNPs and their Composites in ECBs

### 3.1. Influence of MNP Morphology in Biosensing

**Figure 13:**
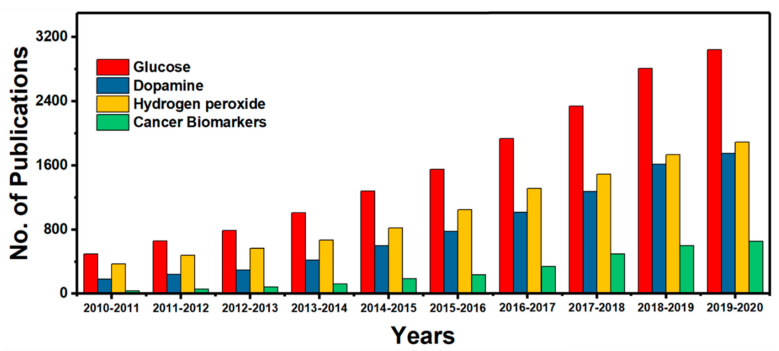
The trend in ECB research over the last decade. The statistical data were obtained from Google Scholar search engine using the following keywords in advance search mode: metal nanoparticle + “glucose”/”dopamine”/”hydrogen peroxide”/”cancer biomarker” + “electrochemical biosensor” for the respective years.

The size and shape of metal nanoparticles plays a crucial role in improving the electrocatalytic activity, selectivity in biomolecular binding, metal–electrode/metal–support interactions for electrochemical biosensing applications [63,82,128,129,130,131]. As a result, research into MNP-based ECBs has received considerable attention. Figure 13 shows the rise in research interest in the field of ECBs from 2010–2020. In this section, the extensively studied MNPs are discussed (Au, Ag, Cu, Pt NPs etc.) based on their size, shape, and structure dependent electronic properties that significantly influence their sensing ability [130,131,132,133]. The synthetic procedures that are regularly utilized to create these MNPs and the technological advancement for characterizing these MNPs are also explored in sequence.

#### 3.1.1. AuNPs: Effect of Size and Shape in Biosensing

AuNPs are the most commonly employed MNPs in ECBs due to their outstanding chemical and electrical properties, excellent biocompatibility, and catalytic ability [54,130,131]. These properties resolutely depend on the size and shape of the AuNPs [133,134]. It is well known that the high index facets and edges of the Au surface are more likely to enhance catalytic activity in contrast to flat or spherical surfaces [135]. Depending upon the synthesis protocols, the properties of the AuNPs, particularly the size and shape, can be precisely controlled. Tremendous efforts have been put forward over the decade to synthesize AuNPs with precise control over size (varying from 1–100 nm) and shape [136]. In a typical wet chemical synthesis process (Turkevich and Lee Miesel process), the metal salt is reduced in the presence of a stabilizing agent or an adsorbate or a capping agent which selectively binds to the surface of AuNPs in order to protect them from aggregation and, therefore, imparting greater stability [137,138]. Furthermore, the controlled nucleation and crystal growth mechanism influence the morphology of the prepared AuNPs and thus allow the formation of different shapes, such as Au nanorods, nanocubes, nanowires, nanopyramids/bipyramids nanocages, nanoflowers, etc. [131,133,139].

**Figure 14:**
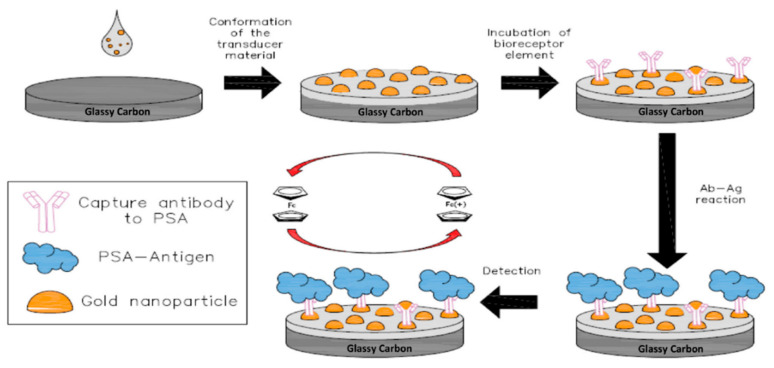
ECB for the detection of prostate specific antigen (PSA). PSA antibody was immobilized on the AuNP–functionalized MWCNT/GCE system that selectively interacted with the PSA–antigen [141]. Reprinted with permission from [141], Copyright © 2020 Published by Frontiers.

In one study, 3D-printed tubes were designed for the simultaneous detection of glucose and H_2_O_2_ [140]. The 3D-printed tube utilized two working electrodes (WEs). One WE was modified with colloidal PtNPs, and the other one with spherical AuNPs and HRP. The colloidal PtNP was utilized for glucose sensing, while the AuNP–HRP system was used for H_2_O_2_ sensing. The sensor showed a broad linear range and low LOD for both analytes, showing the effectiveness of MNP shape control in biosensing ability [140]. The effect of AuNP size on the effectiveness of ECB was investigated by Quintero-Jaime and coworkers [141]. AuNPs were impregnated on functionalized MWCNTs (fMWCNTs) in a ratio of 0.5 and 50. The AuNP–fMWCNT-0.5 ratio system showed AuNPs of 9.5 nm, and the AuNP–fMWCNT-50 ratio system showed AuNPs of 6.6 nm. Based on the size of AuNPs, the prepared ECB showed different linear range and sensitivity for PSA detection. The AuNP–fMWCNT-0.5 ratio and AuNP–fMWCNT-50 ratio systems showed linear ranges from 0–4 ng/mL and 0–6 ng/mL, respectively [141]. The sensor fabrication process is shown in Figure 14.

#### 3.1.2. AgNPs: Effect of Size and Shape in Biosensing

Besides being highly conducting and biocompatible, AgNPs are also one of the most commonly manufactured MNPs [54,142]. They are more easily oxidized compared to AuNPs in an electrochemical setup [19,26,54,143]. This makes them an excellent candidate for ECBs. Despite these advantages, the use of AgNPs is limited by the fact that they are less stable and cannot easily be functionalized [54,144]. Contemporary research in AgNP synthesis for biosensing processes is aimed towards eliminating these limitations. As a result, various methods have been developed for size- and shape-controlled stable AgNPs synthesis [145].

AgNPs of definite size can be produced through the chemical reduction process. The same method that Turkevich and coworkers developed for spherical AuNPs synthesis can also be used for AgNPs [54,137]. AgNO3 is the most commonly used metal salt due to its good solubility in polar solvents. The size of synthesized AgNPs can be controlled through the use of an appropriate reducing agent. Citrate usually produces AgNPs between 50–100 nm diameter, while 5–20 nm AgNPs are obtained when NaBH4 is used [146,147].

Morphological properties of AgNPs can strongly influence its applicability in electrochemical sensing applications. For instance, an MWCNT–AgNPs modifier was shown to be able to detect glucose from 0.025 to 1.0 mM when incorporated on a GCE with GOx [148]. The ECB followed the first-generation mechanism of dissolved oxygen reduction for glucose [9,53]. The average size of AgNPs was determined to be around 5 nm for this system [148]. It has been previously reported that the oxidation potential of AgNPs shifts towards a more negative potential with decreasing size [128]. The thermal scattering is also accelerated in AgNPs smaller than 5 nm [129], suggesting that the small size of the AgNPs played a crucial role in dissolved O_2_ reduction process. 

#### 3.1.3. PtNPs: Effect of Size and Shape in Biosensing

Besides gold and silver NPs, platinum NPs (PtNPs) are also frequently employed in ECB fabrication [149,150]. PtNPs are highly conductive, relatively stable, and have good catalytic activity [150]. Aside from these benefits, PtNPs can catalyze hydrogen peroxide (H_2_O_2_) decomposition during an electrochemical process [151,152]. This is an important property because it can work in synergy with enzymatic processes to significantly amplify the electrochemical current response while lowering the overpotential requirement [152]. The electrocatalytic activity of PtNPs is also dependent on structural properties.

As is the case with other MNPs, it is possible to prepare PtNPs that have a definite size and shape. The most commonly employed synthesis strategies include chemical reduction, electrochemical reduction, electrodeposition, and the photochemical reduction of platinum salts (PtCl_6_^2−^ and PtCl_4_^2−^) [152].

**Figure 15:**
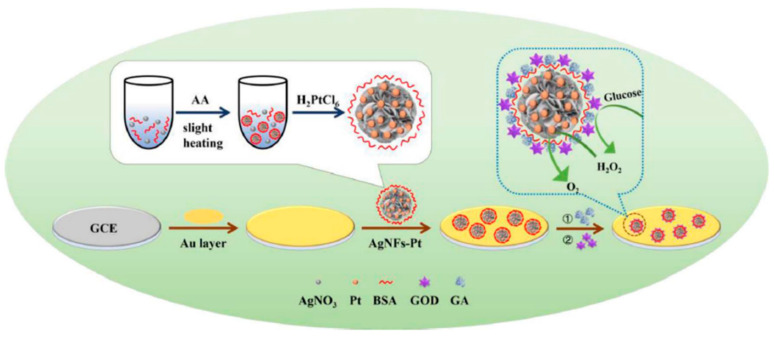
Fabrication of an ECB for the simultaneous detection of glucose and H_2_O_2_. The sensor utilized an Ag nanoflower and PtNPs (AgNFs-Pt) along with enzymes to be able to detect two analytes simultaneously [153]. Reprinted with permission from [153], Copyright © 2020 Published by ECS. AA: L-ascorbic acid.

PtNPs with cubic, polygonal, or rod shapes offer better anchoring sites for biorecognition components compared to spherical NPs [151]. Huang et al. reported the development of a highly effective glucose and H_2_O_2_ ECB [153]. For this, flower-like AgNPs were decorated with dewdrop-like PtNPs for enhancing the electrocatalytic surface area, selectivity, and stability. Figure 15 shows the synthesis and morphological structures of the ECB. The sensor showed linear range from 1 µM to 2 mM for H_2_O_2_ and 1 to 14 mM glucose [152].

### 3.2. Properties of MNPs Composites

MNPs have excellent electrocatalytic activity. However, when used in biosensing application these properties need to be fine-tuned [153,154]. The most convenient way of tuning the properties of MNPs is through combining MNPs with other conducting nanomaterials (CNMs). Tran and coworkers reported a graphene quantum dot (GQD) and AgNP nanocomposite for detection of glucose [155]. The composite showed a wide linear range of 1–10 mM, although the composite had larger size (~40 nm) compared to previously reported NPs [155]. Because of their ability to work synergistically, CNMs such as fullerenes, GR, rGO, quantum dots (QDs), calixarenes, and carbon nanotubes (CNTs) are frequently employed for preparing MNP composites (MNPCs) [156,157]. The electrocatalytic activity of these MNPCs is largely dependent on the choice of MNPs and CNMs [154]. Hence, it is crucial to have a sound understanding of the physical, chemical, and electrical properties of these CNMs before using them as electrocatalysts in preparing ECBs.

#### 3.2.1. Fullerene-Based MNPCs

Since their discovery in 1985, these sp^2^ carbon-containing truncated icosahedron-shaped fullerenes have found practical use in a wide range of applications [158]. The C60 and C70 fullerenes can be reduced in a reversible manner for up to six electrons (1e^−^ each step) transfer process in nonaqueous solvents [159]. The electrooxidation of fullerenes is often irreversible in nature [159]. The ease of electrooxidation or reduction is dependent on the size and symmetry of the fullerenes [158,159]. Usually, larger fullerenes are more electroactive [160]. Fullerenes can be easily functionalized through such redox processes. Furthermore, fullerenes can be synthesized in a way that they interact with cations, metal atoms, or small molecules via coordination or adduct formation to form endohedral and exohedral systems [159]. Electron spin resonance (ESR) analysis showed that the metals in endohedral systems are typically in the oxidized form, making the fullerene skeleton negative charge bearers. These endohedral fullerenes act like organic salts, meaning these are capable of interacting with both positively and negatively charged systems. As a result, biorecognition systems carrying opposite charges can be utilized with ease for preparing biosensors [159,161]. Compton et al. first reported fullerene-modified electrodes for sensor application. MNPs can be incorporated with fullerenes through both endohedral and exohedral means for preparing ECBSs [159]. 

#### 3.2.2. CNT-Based MNPCs

CNTs are a class of nanostructured CNMs that are of tubular shape with few nanometers in diameter, and lengths in the micrometer range [161]. These are either single wall CNTs (SWCNTs) or MWCNTs, based on the number of rolled-up layers [158]. Some of the unique properties of CNTs are excellent electrical and thermal conductivity, good elasticity (~18%), high tensile strength and flexibility, and good biocompatibility [118,152]. It has been shown that synthetic cardiac cells can be grown on CNTs without any significant toxic effects [162]. Besides, both SWCNTs and MWCNTs can be functionalized to facilitate binding with biorecognition entities through electrostatic interactions or covalent linkages for fabricating effective ECBs [141,163].

#### 3.2.3. GR-Based MNPCs

**Figure 16:**
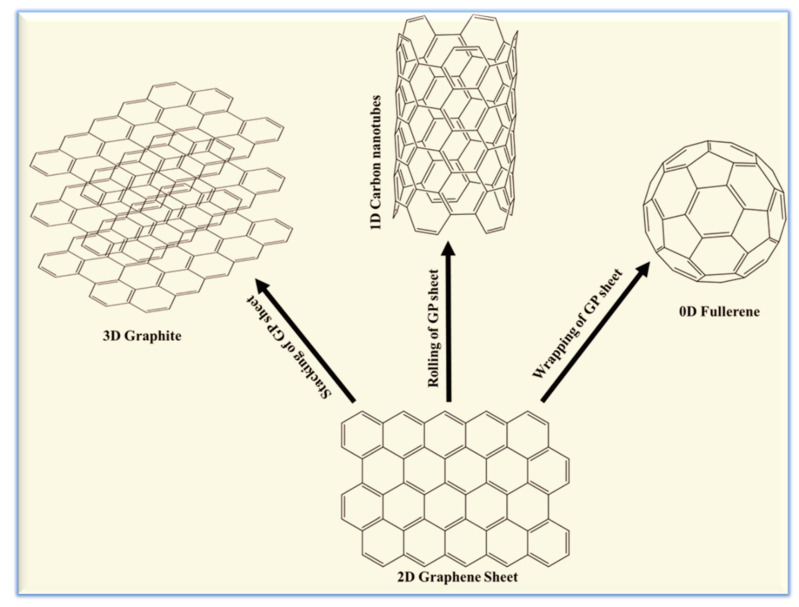
Schematic presentation of the relationship between different allotropes of carbon [9]. Redrawn from [9].

Single sheet GR (graphene) was first prepared through mechanical exfoliation by Geim and coworkers in 2004 [9]. Of all the allotropes of carbon, the electronic properties of GR are the most fascinating. GR can be considered in biosensing applications as single layer GR (SLGR), few layer GR (FLGR), and multilayer GR (MLGR) [164]. SLGR is crystalline in nature, FLGR is crystalline up to 10 layers, and beyond that 3D graphite-like properties are observed [9,156]. SLGR sheets have very high conductivity (~1.6 × 10^5^ S/cm) and low resistance (30 Ω/sq) [9]. The anharmonic stretching and bending vibrational modes of sp^2^ hybridized GR sheets are responsible for forming the finite “rippled” structures that stabilize the 2D sheets while promoting unique electronic properties that are not observable in other allotropes of carbon [165]. These properties include the absence of a weak localization force, ambipolarity of charge carrier concentration, and ballistic electron transport [166,167]. GR can be derivatized for producing graphene nanoribbons (GRNs), GO, rGO, GR nanowalls (GRWs), and GQDs [9]. Each of these derivatives have their own electronic properties that enables them to be used in diverse biosensing platforms [155]. GR sheets can be considered as the building block for the other allotropes of carbon. Figure 16 shows a schematic representation of how CNTs, fullerenes, and graphite are related to GR.

Table 1 discusses MNPC-based ECBs that have been reported for the detection of various biomolecules based on the size and shape of MNPs. It can be seen from the table that MNPs with sizes between 5 and 20 nm are the most-used individual component in the ECB fabrication process [4,23,26,27,29,41,44]. Although size variation is well studied, the shape of the MNPs used is almost always spherical [4,26,41,44,48,85]. The area of MNP shape control deserves significant attention, because NPs with unique shapes (hollow spheres, cubic, porous, pyramidal, etc.) are likely to offer better catalytic activity, increased surface area, and enhanced stability for the ECBs [23,28,39].

## 4. MNP-Based ECBs for Biomolecule Detection

The previous section discussed the properties of MNPs and MNPCs that influence their effectiveness in ECBs. This section will discuss about the advances in ECB design strategies for the rapid and effective detection of small biomolecules (SBMs), cancer biomarkers, and the COVID-19 virus.

### 4.1. MNPs in Small Biomolecule Sensing

SBMs are organic compounds that do not have extended polymerization and are responsible for maintaining chemical balance throughout the body [171,172]. In the case of diabetes, the world’s ninth deadliest disease, the blood glucose level can change drastically (up to 30 mM) from the normal level of 4 to 8 mM [173,174]. Dopamine (DA) is a neurotransmitter that is responsible for controlling the motor and sensory nerves, feelings, and various other body functions [22,175]. Abnormality (normal level 0.01–1 µM) in DA level is responsible for attention deficit hyperactivity disorder, Alzheimer’s disease, and schizophrenia [175]. Furthermore, the irregularity in DA level can be used for determining Parkinson’s disease and HIV [175,176]. Uric acid (UA) is the end product of purine metabolism that is normally present in the body within 100 µM concentration range [176]. It helps to relieve stress caused by oxidative processes. However, in excessive content it can cause gout and hyperuricemia [176]. During the production of antioxidant UA through the xanthine oxidation process, H_2_O_2_ is formed as a short-lived oxidizing agent that can show acute toxicity [177]. Usually, only a trace amount of H_2_O_2_ (10–100 nM) is found in the circulated blood, which makes detection an arduous task [177]. While glucose detection requires a wide linear range, other SBMs necessitate a highly sensitive approach. ECBs that are used in the detection of SBMs are often biocatalytic in nature and utilize impedimetric, amperometric, and voltammetric EATs [129,154].

**Figure 17:**
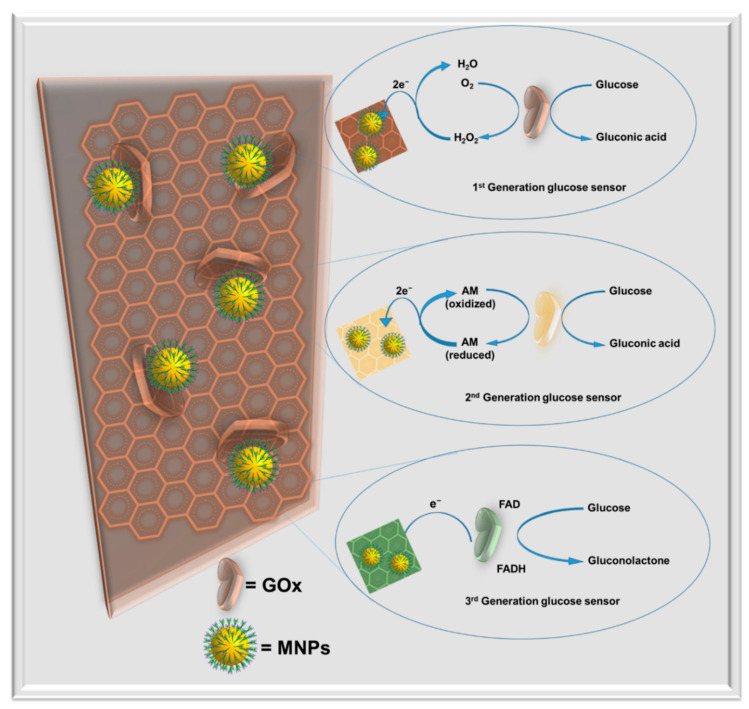
Mechanism of glucose oxidation at ECBs that utilize biocatalytic system. The mechanisms for 1st, 2nd, and 3rd generation glucose sensors are shown [9]. Redrawn from [9].

**Figure 18:**
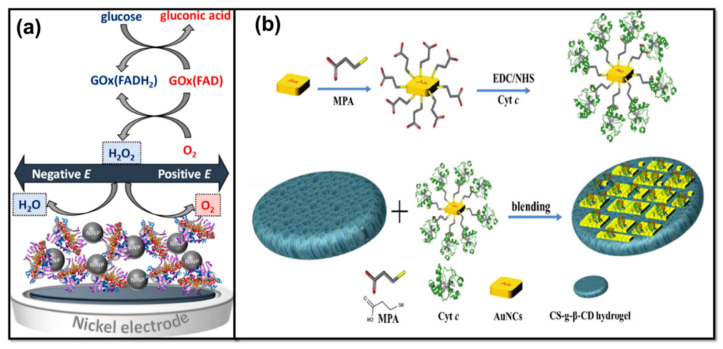
Electrode fabrication process and detection of glucose and H_2_O_2_. (**a**) the possible mechanism for the simultaneous oxidation of H_2_O_2_ and glucose molecules by NiNP/Ni substrate-based enzymatic ECB [179]. (**b**) The synthesis process for cyt c conjugated AuNCs imbedded hydrogel ECB for the sensitive detection of H_2_O_2_ [182]. Reprinted with permission from [179], Copyright © 2020 Published by the American Chemical Society [182], Copyright © 2020 Published by Elsevier B.V.

GOx is one of the most frequently used enzymes for the glucose detection ECBs, because it is capable of inducing a direct electron transfer through its two flavin adenosine dinucleotide (FAD) coenzymes [9]. The enzyme-based glucose sensors are categorized as: (i) first generation ECBs that utilize O_2_ molecules as mediators to oxidize FAD to FADH_2_; (ii) second generation ECBs that use artificial mediators for glucose sensing; (iii) third generation ECBs that induce direct electron transfer between glucose and the immobilized enzymes [9]. The mechanisms of these three types of glucose sensors are shown in Figure 17. MNPs can significantly enhance the performance of enzymatic glucose sensors through providing a high surface area, alternative low energy catalytic pathway, and stability for immobilized enzymes [9]. A PtNP-coated SnS_2_ enzymatic (GOx) glucose sensor was reported with linear range from 0.1–12 mM [168]. Authors concluded from morphological analysis of the prepared electrochemical glucose biosensor that the use of hydrophilic PtNPs significantly enhanced GOx immobilization. This in turn resulted in the sensitive detection of glucose over the wide linear range [168,178]. Magnetic NiNPs have been used for directly immobilizing GOx [179]. The glucose sensor showed linearity up to 12 mM with an LOD of 0.42 mM. The proposed sensing mechanism for the sensor is shown in Figure 18a. The magnetic NiNP sensor did not need to incorporate any other binding material for GOx immobilization [179].

Although HRP is most commonly employed for H_2_O_2_ detection, other redox-inducing biorecognition components such as ferredoxin, cytochrome C, and hemoglobin are also utilized [180]. A myoglobin-based H_2_O_2_ sensor was reported which used MoS_2_ NPs and GO [176]. The myoglobin/MoS_2_ NP/GO system showed the best current response along with better stability compared to only myoglobin/MoS_2_ NPs or myoglobin/GO systems [181]. In another study, cytochrome C enzyme was used for fabricating the H_2_O_2_ sensor—Au nanocubes were utilized for immobilization of cytochrome C [182]. Figure 18b shows the fabrication process of the reported H_2_O_2_ ECB. The sensor showed a linear range from 100–1000 µM for H_2_O_2_ detection [182]. The use of cubic NPs enhanced the electroactive surface and incorporated a better electron transfer mechanism for biocatalytic H_2_O_2_ reduction. This work shows the importance of shape- and size-controlled MNP fabrication for use in the ECBs.

Unlike H_2_O_2_ and glucose, there is no specific biocatalyst that is employed for the detection of DA. Paulraj and coworkers showed that polyaniline (PANI)-coated AgNP-modified electrodes can be used for simultaneously detecting DA and H_2_O_2_ [178,183]. The sensor was utilized for oxidizing DA and reducing H_2_O_2_. The proposed sensor showed a low LOD of 0.03 and 0.12 µM for H_2_O_2_ and DA, respectively [178,183]. However, UA is often detected with the help of uricase (UOx) enzyme [182,184]. A Cu2ZnSnS4 NP-modified ECB was reported for the detection of UA [182,184]. The sensor utilized UOx in combination with the Cu_2_ZnSnS_4_ NPs for the detection of UA. The low LOD of 0.066 µM and wide linear range of 50 to 700 µM shows the effectiveness of using UOx enzyme in the modification process [182,184]. In another work, an AuNP and rGO complex was utilized for the immobilization of UOx [183,185]. This biosensor was utilized for the rapid detection of UA from human serum samples. The sensor required lower positive potential (low overpotential) compared to traditional electrochemical sensors. The linear range was from 50 to 800 µM with good selectivity and real sample analysis results [183,185]. These works show the advantage of using ECBs compared to traditional methods in the detection of small biomolecules. The high sensitivity, good selectivity, ease of preparation, rapid detection, and cost-effectiveness are the most attractive aspects of these ECBs. Table 2 discusses the fabrication process and EATs utilized for the detection of small biomolecules.

### 4.2. MNPs in Cancer Biomarker Detection

Carcinogenesis happens at a genetic level in the cell, and follows a complex pathway that ultimately disturbs the homeostatic equilibrium by altering the cell death and cell proliferation rate [185]. Despite tremendous efforts and development, treatment of cancer is still challenging because of the following reasons: (i) proliferation of cancer cells and cell proteins through mutation of the proto-oncogenes; (ii) rejection of growth inhibition signals; (iii) evasion of apoptosis or activating anti-apoptotic genes in cells [69,190,191]. For early-stage diagnoses of cancers, tracking of the disease-specific biomarkers is essential. Biomarkers are characteristics biomolecules overexpressed in the beginning of carcinogenesis, either by the body immune system in response to the disease or by the tumor cell itself [11,71,97,100]. Biomarkers could be utilized to assess the responses from the body towards a specific treatment process for controlling disease [191]. A wide range of biomarkers based on genetic, proteomic, glycomic, etc., are well established for detecting cancers and the prognosis processes [192,193,194]. Evaluation of these different biomarkers in bodily fluids such as serum, blood, urine, saliva, tears, and sputum would require noninvasive and cost-effective methods for cancer screening [191,192,193,194]. As a result, biomarker detection based on electrochemical methods has been perceived as an effective early-stage diagnosis of cancer, even though clinical sampling and analysis is still in its infancy. The type of materials utilized for electrode modification are mostly MNPs and MNPCs. This has been discussed in detail in Section 3. MNPs provide improved biocompatibility, better surficial stability, and binding affinity for biomolecule conjugation [82,105,130]. Such surface immobilization of BRCs (antibody, peptide, or aptamer) for preparing immune/aptasensors depends on the functionality of the biomolecule and the type of nanostructured electrode modifiers used. Both of these need to be compatible with each other [62,155,179,183]. For example, AuNPs allow thiol-functionalized antibody/aptamers to be anchored over the electrode surface via activation of the thiol group (SH)–Au bond, which is one of the fundamental pathways followed in most electrochemical bioaffinity sensor preparations [97,152]. In short, bioaffinity ECB functions are based on such binding capacities of the BRCs via interactions with the nanostructured electrode materials. The BRCs are mainly antibodies (mono/polyclonal), aptamers (single stranded ssDNA sequence/RNA), peptides, etc., which can effectively capture target antigens or biomarkers while constructing bioaffinity ECBs [44,85,98,155]. Table 3 shows the mostly studied bioaffinity ECBs for different biomarkers based on MNPs and nanocomposites assays.

#### 4.2.1. MNPs in Carcinoma Embryonic Antigen Sensing

**Figure 19:**
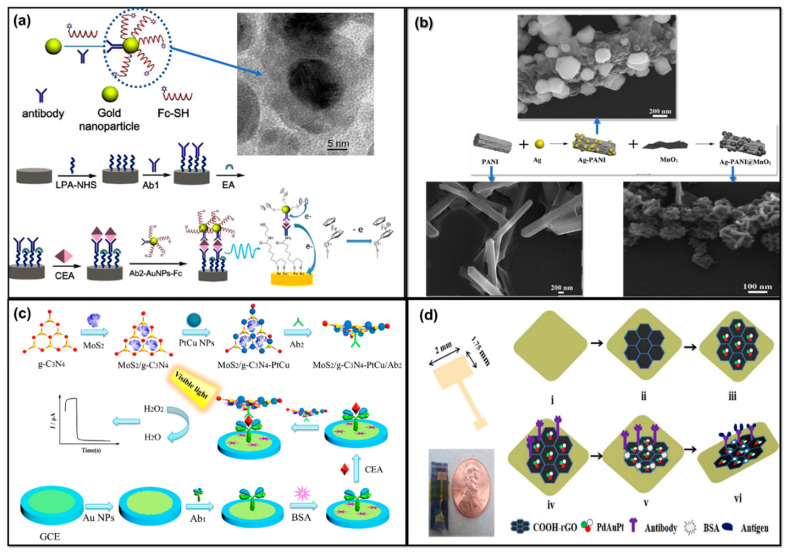
ECBs for the detection of CEA. (**a**) Schematic illustration of two step synthesis process involved in the fabrication of sandwich immunosensor. The TEM image shows Anb_2_- and Fc-labeled AuNPs [197]. (**b**) The synthesis step for Ag–PANI@MnO_2_. The SEM images show PANI, Ag–PANI, and Ag–PANI@MnO_2_ [201]. (**c**) Fabrication of MoS_2_/g–C_3_N_4_–PtCu bimetallic sandwich immunosensor system for light-enhanced CEA detection [199]. (**d**) Development of a trimetallic (Pd@Au@Pt) ECB and picture of the final strip sensor with a coin for comparison of the size [195]. Reprinted with permission from [195,197], Copyright © 2020 published by Elsevier B.V. [199], Copyright © 2020 published by Springer Nature [201], Copyright © 2020 Hydrogen Energy Publications LLC. Published by Elsevier.

Carcinoma embryonic antigen (CEA) is a cell adhesive acidic glycoprotein with properties similar to the human embryonic cell. Normally, the level of CEA is around 5 µg/mL in serum, but in the blood the level is very low (<5 ng/mL) [195,196,197,198]. Meanwhile, serum CEA has been found to elevate up to 20 µg/mL in people with lung cancer [197]. Blood CEA levels above 10 ng/mL are indicative of cancer in the patient [201]. In several other types of carcinomas, such as breast cancer, ovarian cancer, pancreatic cancer, and gastrointestinal cancer, CEA often shows elevation in serum level, which indicates its potentiality as being a tumor marker for clinical cancer diagnosis [195,196,197]. Therefore, quantitative measurements of CEA in biological fluids such as blood and serum are critical for locating, and understanding the prognosis, staging, and recurrence of multiple cancers [197,198,199,200,201]. For electrochemical immunosensor/aptasensor assays, MNP-based probes which have strong biocompatibility and electrical conductivity are of great interest [201,202,203,204,205]. MNPs have superior efficiency as tags or labels for amplifying biomolecular interactions and as the enhancers of electrochemical signals [198,199,200,201,202,203]. Depending upon the complexity and necessities, mono/bi/tri-metallic composites are utilized for constructing different types of ECBs with label-free or labeled, sandwich or non-sandwich strategies for CEA immunosensor/aptasensor [195,196,199,207,210]. In mono-metallic-based ECBs, the primary concern is to enhance electrocatalytic surface area for anchoring biomolecules such as the antibody, and labeling enzymes or conductive dyes or biomolecules for developing labeled immunosensors [197,201]. Normally, direct immobilization of electrical signaling molecules such as HRP, MB, Fc to MNPs is not beneficial because it causes the loss of signaling molecules during electrochemical experiments and hence poor stability and reproducibility of the sensor [197]. To overcome this problem, Gu et al. constructed an Fc-labeled AuNP-based sandwich immunosensor assay for the highly sensitive electrochemical detection of CEA [197]. In this work, they introduced a thiol group (-SH) into an Fc molecule which assisted the stable chemisorption of Fc over AuNPs via initiating an S-Au covalent bond. After immobilization of Fc–SH, the colloidal Au nanoprobes were stabilized with PEG800. Figure 19a shows the transmission electron microscope (TEM) image of the nanostructured Fc–SH/AuNP–Ab_2_ composite along with the schematics of the fabrication process. Through this Fc-labeled immunosensing assay, they achieved a detection limit for CEA as low as 0.01 ng/mL [197]. Apart from AuNPs as the effective sensing platform and capture antibody, AgNPs also showed great promise in enhancing conductivity for electrocatalytic sensing and constructing immunosensors. Zhao et al. utilized a dual reduction signal amplification strategy based on AgNPs and MnO_2_ for constructing a sandwich immunosensing assay for CEA [201]. AgNPs and MnO_2_ together displayed catalytic activity for the reducing of H_2_O_2_ into H_2_O and molecular O_2_. At the same time, they utilized PANI, which acted as the sacrificial reducing agent for AgNP and as the base material for providing active sites for AgNP and MnO_2_ immobilization. Figure 19b shows the SEM images of the composites at different fabrication stages. This ultrasensitive dual amplifying sandwich immunosensor showed an LOD for CEA of about 0.17 pg/mL with a broad sensing range of 0.0005–80 ng/mL [201].

In bimetallic-based ECBs, synergistic interactions between multiple MNP components might be able to induce significant signal amplification, when compared with mono metal components [199,215]. In addition, the bimetallic counterpart shows enhanced photo-induced charge transfer properties and biocompatibility [199]. For instance, Song et al. constructed an MoS_2_/C_3_N_4_ (graphitic) composite supported with bimetallic Pt–Cu nano-dendrimers for visible light-induced amperometric sandwich immunosensing assay for CEA detection [199]. Graphitized carbon nitride usually has low conductivity due to its compact conduction band. However, by compositing with 2D MoS_2_, interactions between the conduction band of C_3_N_4_ and valence band of MoS_2_ enhanced the catalytic and charge transfer capacity of the support for bimetallic Pt–Cu NPs. The synergistic charge transfer/flow efficiency of the bimetallic counterparts along with the conductive support allowed for visible light-induced increment of the amperometric current signal for H_2_O_2_ reduction after the formation of the sandwich immunosensing array [199]. Figure 19c shows the synthesis process of the bimetallic ECB. In another work, monodisperse flower morphology-based Pt–Au NPs-supported rGO composite was prepared through gamma irradiation followed by microemulsion strategies for constructing a pulse voltammetric ultrasensitive CEA immunosensor [215]. Gamma irradiation ensured the simultaneous reduction of both GO and Pt–Au NPs, while water droplet-based microemulsion controlled the unique flower-like morphology and the size of the bimetallic Pt–Au NPs (<15 nm). The low LOD for CEA obtained using this immunosensor was only 7 fg/mL [215].

Trimetallic nanoparticles as the electrode probe are even more attractive in a sense of fast electron transfer affinity and great electrode stability when compared to the mono/bi-metallic counterparts [195]^.^ Their finite geometrical orientation of the metallic components and HOMO (Highest occupied molecular orbital) -LUMO (Lowest unoccupied molecular orbital) distribution due to the formation of mixed bonds with the help of ligands enables formidable synergistic electrocatalytic activity. Barman et al. constructed a trimetallic Pd–Au–Pt NP-supported COOH functionalized rGO composite for developing an immunosensor for both CEA and PSA detection [195]. Figure 19d shows a schematic representation of the trimetallic sensor fabrication process. In this report, CV-assisted electrochemical deposition of trimetallic composites over COOH–rGO-modified gold electrode was implemented because the composition and morphology of the MNPs can be tailored by precisely controlling the concentration of the precursor metal salt solution, pH, scan rate, CV cycle, and deposition potential. The composite was successfully utilized to anchor the capture antibody and subsequently the antigen. The sensor delivered an ultrasensitive response toward CEA with an LOD of 8 pg/mL [195]. The authors emphasized that the sensor could be used for the POCT of CEA from human serum. Aside from MNP–carbon material-based composites, metal oxide NPs, core shell MNPs, quantum dot NPs, graphene, MOF, etc., have extensively been studied as the electrode probes for fabricating both labeled or label-free sandwich immunosensing arrays for the ultrasensitive detection of CEA for early-stage cancer diagnoses [196,202,209,211,212,213,214,215,216].

#### 4.2.2. MNPs in Prostate-Specific Antigen Sensing

Prostate cancer (PSC) is one of the most common cancers for males with high mortality [219]. At present, prostate cancer can be treated by removal of the cancerous cells, but only if diagnosed in the early stages [219,220,221,222,223]. However, in the advanced stages, PSC cancer is lethal. Research into prostate cancer has shown that the early stage of the cancer is asymptomatic [223,224,225]. Hence, sensitive and selective detection of PSC biomarkers is most desired for early-stage detection. Prostate-specific antigens (PSAs) are one of the most reliable biomarkers for the early-stage detection of PSC [220,221]. Previous research has shown that a presence of PSA between 4–10 ng/mL is indicative of the possible PSC (>27%) risk [225]. If the PSA level is 10 ng/mL or above, then the risk is increased to greater than 67% [222,225]. As a result, the PSA biomarker is used for monitoring both the progression of the prostate cancer and mediating therapeutics [220]. Hence, significant research effort has been put into the development of ECBs for the detection of PSA.

**Figure 20:**
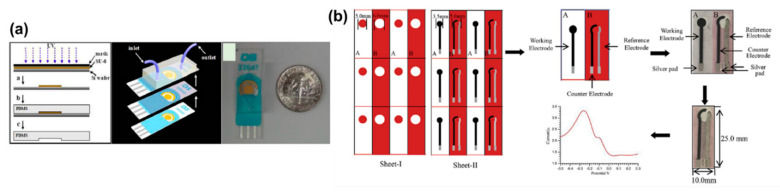
ECBs for the sensitive detection of PSA and cancer antigen 125 (CA125) biomarkers. (**a**) schematics for the fabrication of a microfluidic PSA sensor through screen printing, and size comparison image [237]. (**b**) fabrication of paper-based ECB for CA125 detection [249]. Reprinted with permission from [237], Copyright © 2020 published by Springer Nature [249], Copyright © 2020 published by Elsevier B.V.

PSA is a glycoprotein of around 34 kDa in molecular weight (MW) [223]. The low level of PSA makes it very hard to detect. As a result, researchers have explored different strategies for designing effective BRCs for PSA sensing. A highly sensitive biosensor was reported to utilize PdNPs along with conducting PANI and fullerene-C_60_ for PSA sensing [219]. The PANI–C60 combination worked towards activating the PdNPs through electrostatic interactions. This in turn allowed for the successful immobilization of the PSA on the sandwich type immunosensor. The sensor reported a promising linear range from 0.00016–38 ng/mL with a level of detection of 1.95 × 10^−5^ ng/mL. The sensor was also tested for the low detection of prostate cancer from serum and urine samples, with a recovery between 95–97% [219]. Another work utilized a screen-printing process to make prostate cancer sensors that could be used for practical applications [225]. The sensor utilized a GO and AgNP composite over screen-printed carbon electrodes (SPCEs) for the immobilization of PSAs. The label-free immunosensor offered a simple preparation electrode process compared to the complicated sandwich sensors. The biosensor utilized a “signal off” methodology for the PSA interaction with the antibody through the DPV technique. Despite the simple preparation process, the sensor showed a wide linear range from 0.75–100 ng/mL [225]. This indicated the feasibility of the screen-printed label-free sensor in POCT. A number of works have reported that bimetallic, MOF, QD, and core@shell structure further enhance the sensitivity and stability of ECBs [220,221,222,224,227,235]. However, as discussed in the previous sections, it is important to be able to prepare ECBs on strips so that they can be used for the on-the-spot testing of biomolecules. To this end, Chen and coworkers reported the fabrication of a PSA biosensor based on the microfluidic principles through screen printing [237]. The ultra-sensitive PSA sensor was prepared through screen printing, making it readily scalable and cost-effective. The sensor fabrication process is shown in Figure 20a. The proposed sensor used printed gold electrodes as the WE and CE, while an Ag electrode was used as the control. MagBs were utilized for anchoring the PSA antibody on the printed gold electrode. The sensor utilized an amperometric technique for the PSA detection from 0.001–10 ng/mL, with a low LOD of 0.00084 ng/mL. Authors claimed that the reported sensor was cheap, easy to fabricate and operate, highly reproducible, and extremely sensitive [237]. Such sensors could be the key to solving the problems associated with utilization of ECBs for the POCT.

#### 4.2.3. MNPs in Cancer Antigen 125 Sensing

Ovarian cancer is one of the most commonly occurring cancers for woman and has high mortality rate [194,247]. The main reason behind the high mortality rate is the fact that early stages of ovarian cancer are usually asymptotic (stage I), and for later stages the symptoms are unspecified (stage II and III) [245]. A promising biomarker for the early-stage detection of ovarian cancer is cancer antigen 125 (CA125) [244,246]. CA125 is the recommended biomarker for ovarian cancer diagnosis by the FDA [194]. CA125 is a mucin-like transmembrane glycoprotein (200 kDa) that is overexpressed even during the early stages of ovarian cancer [245]. For a healthy person, the CA125 level is below 35 U/mL in blood and serum [245]. However, the level of CA125 in the blood and serum increases significantly for patients with ovarian cancer. That is why CA125 is known as a “Gold Standard” biomarker for ovarian cancer diagnoses [287]. CA125 overexpression is observed in about 50% of stage I patients, and about 90% overexpression is observed for stages II, III, and IV patients [244,245]. Therefore, a sensitive, selective, and accurate POCT system for CA125 is essential for the early-stage diagnosis and treatment of ovarian cancer.

The demand for early-stage detection of ovarian cancer has led to the development of a large number of ECBs for CA125. AuNP-modified biosensors have been the most reported for CA125 detection [244,246,248,249,251]. These works have utilized various CNMs for increasing the selectivity and sensitivity. For instance, AuNP, PAMAM, MWCNT, and rGO composites were utilized for immobilizing the antibody (Anb1) [244]. Here, MB was used to label the Anb_2_. This sandwich (Anb_1_–Ang–Anb_2_) system utilized the SWV technique for CA125 detection. The sensor showed a moderate linear range from 10–75 U/mL with a low LOD of 0.006 U/mL [244]. In another study, Huang et al. utilized an AgNP-MagBs-based sandwich immunosensor system for CA125 detection over a wide linear range of 0.001–76 U/mL [246]. The electrochemical cell setup utilized a magnet-controlled microfluidic flow system and the LSV technique for the detection process. The proposed sandwich system showed significant signal enhancement and selectivity towards CA125 detection [246]. Although these sensors have potential, it is often difficult to transition from laboratory to clinical applications. A paper-based strip ECB design was used to address this issue [251]. The proposed sensor utilized AuNP, rGO, and thionine composite as the substrate for immobilization of the CA125 antibody to fabricate the “signal off” ECB. The immunosensor utilized the affinity-based binding between the CA125 Anb–Ang to decrease the current signal produced by the thionine. The fabrication and detection of CA125 is shown in Figure 20b. As a result, the current signal decreased with increasing concentration of the CA125. The sensor showed a linear range from 0.01 to 200 U/mL for CA125 [251]. Development of these strip sensors might solve the problems associated with the clinical transition of ECBs.

#### 4.2.4. MNPs in HER2 Sensing

One of the most prominent candidates for early-stage breast cancer detection is the HER2 [262,263]. This protein expression status is either positive or negative for the cancer [257]. It is often overexpressed in the early stages of breast cancer [262]. Breast cancer is one of the most frequently observed (~34%) cancers in all woman [263]. HER2 concentration in the blood of a healthy human body is about 2–15 ng/mL [288]. However, in a cancer patient the HER2 protein increases to 15–75 ng/mL [263]. The increase in HER2 concentration is significant, which makes it a prominent candidate for the early-stage cancer detection. However, the very low concentration of the biomarker makes it difficult for effective detection using conventional ECBs. This is why researchers have focused on using MNP-incorporated immunosensors, aptasensors, and affisensors for the sensitive and selective detection of HER2 [100,262,263,264,265].

**Figure 21:**
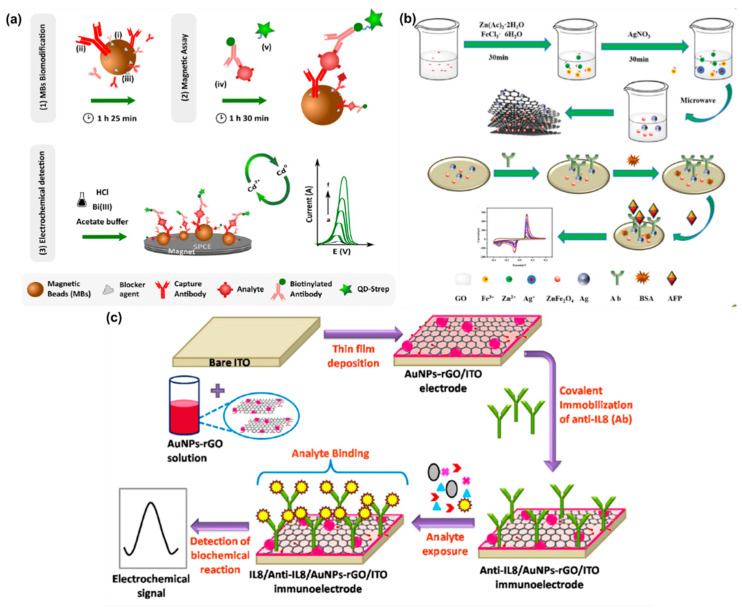
Fabrication and detection mechanism of ECBs for HER2, α-fetoprotein (AFP), and IL-8. (**a**) schematics for the detection of HER2-labeled with CdSe@ZnS QDs through a MagB system [262]. (**b**) synthesis and mechanism of label immunosensor for the sensitive detection of AFP [278]. (**c**) step-by-step synthesis procedure and the selective detection of IL-8 at an AuNPs–rGO composite system [286]. Reprinted with permission from [262], Copyright © 2020 published by Springer Nature, [278], Copyright © 2020 published by Elsevier B.V. [286], Copyright © 2020 published by the American Chemical Society.

Two ECBs that utilized AuNPs and CdSe@ZnS QDs were reported for the selective detection of HER2 [261,262]. The AuNP-modified electrode used a label-free immunosensor and the DPV technique [261]. The sensor showed a low LOD of 0.035 ng/mL, but the linear range was also very short, 0.001–20 ng/mL [261]. The CdSe@ZnS QD sensor had a longer linear range of 0.5–50 ng/mL, with an LOD of 0.29 ng/mL [262]. This ECB utilized functionalized MagBs for anchoring the BRC. The sensor could detect HER2 from the SK-BR-3 (an HER2-positive cell line) for only 2 cells/mL [262]. The detection process is shown in Figure 21a. Gold nanorods (GNRs) and Pd composite electrodes were proposed by Chen and coworkers for fabricating an HER2 biosensor with wide linear range [268]. The ECB used super structure aptamers for the detection of HER2. The sandwich type sensor mechanism pathway was followed, utilizing the DPV technique. A wide linear range from 10–200 ng/mL was obtained with an LOD of just 0.15 ng/mL [268]. Such an HER2 sensor could be used for POCT and diagnosis of breast cancer.

#### 4.2.5. MNPs in Alpha Fetoprotein Sensing

Alpha fetoprotein is a glycoprotein that can be utilized for the early-stage diagnosis of hepatocellular carcinoma (HCC) [272,273]. HCC is one of the most common types of liver cancer that often leads to the death of the patient [273]. Alpha fetoprotein is about 70 kDa in MW, and is produced in the yolk sack, liver, and gastrointestinal tract [272,277]. During cancerous conditions, the alpha fetoprotein concentration is above 500 ng/mL in the human body, while in a healthy body it is only around 20 ng/mL [273,274]. This large abnormality in concentration makes alpha fetoprotein a significant marker for HCC detection. However, it is a challenging task to prepare biosensors for the detection of a low concentration analyte with a wide linear range.

An ECB was proposed that utilized an AuNP–GRN composite and a DPV technique for alpha fetoprotein detection [272]. The label-free immunosensor used Anb–alpha fetoprotein modification for the detection of alpha fetoprotein. It had a low LOD of 1 ng/mL and a linear range of 5–60 ng/mL. Though the LOD is low, the linear range was very small for the proposed senor for POCT of alpha fetoprotein in cancer patients [272]. To address the challenge of wide linear range, Li et al. developed an ECB utilizing AuNPs that had a wide linear range from 0.1 to 100 µg/mL [273]. However, the LOD was 92 ng/mL, which indicates that this sensor could not be used for the low-level detection of alpha fetoprotein. A label-free immunosensor was developed using AgNPs and an rGO composite along with ZnFe_2_O_4_ for the sensitive detection of alpha fetoprotein [278]. The signal amplification of the sensor enabled the CV technique to be used for detecting the pg/mL analyte. The alpha fetoprotein sensor development process and consequent detection mechanism is shown in Figure 21b. The sensor showed a very low LOD of 0.98 pg/mL, with a linear range in the region of 0.001–200 ng/mL [278]. A possible way to improve alpha fetoprotein sensors can be considering the size- and shape-dependent properties of MNPs. At the same time, utilizing different CNMs for preparing MNPCs is a promising way for further improving the biosensing capability.

#### 4.2.6. MNPs in Interleukin Sensing

The human genome has about 50 different small proteins or cytokines that are responsible for maintaining important immunomodulatory responses [289]. Hence, interleukin (IL) is a term that describes this group of cytokines that are capable of being signaling cells. Of the various IL species, in this section the focus will be on IL-6 and IL-8, which are important biomarkers for colorectal and oral cancers, respectively [282,284]. IL-6 contains 184 amino acids that iss about 26 kDa in MW [284]. This glycoprotein is responsible for signaling cancer cells, and overexpression is related to colorectal cancer [282]. On the other hand, the MW of IL-8 is about 8.5 kDa, with its 70 amino acids [284]. Enzyme-linked immunosorbent assay (ELISA) and a few other methods are commercially available for the detection of these biomarkers [285,290]. However, one ELSIA kit costs about USD 320 [291], whereas ECBs can be prepared for much less. Sandwich and structure switching type aptasensors were used for the sensitive and selective detection of IL-6 [282,283], utilizing AuNPs and the impedimetric technique for the detection process. These sensors had an impressive LOD of 0.0016 and 0.00033 ng/mL for IL-6 [282,283]. These reports demonstrate the significance of using MNPs in ECBs. There are various ECB designs for the detection of IL-8. Normally, only 250 pg/mL of IL-8 is present in a healthy person’s saliva [285,286]. IL-8 expression higher than 750 pg/mL is indicative of oral cancer [285,286]. ECBs for IL-8 detection have used Ag2MoO4 and CdTe/CdS QDs, besides AuNPs [285,286]. These multimetallic NPs with a controlled size and unique shapes often perform better than spherical monometallic systems. The multimetallic ECB fabrication is shown in Figure 21c. However, the use of EATs and other supporting ingredients of the biosensors also play an important role in the overall performance of the sensor. The DNA templated CdTe/CdS QD sensor had a very low LOD of 3.36 × 10^−6^ ng/mL for IL-8 detection [285]. The AuNP sensor with the setup of anti-IL8/AuNPs–rGO/ITO showed an LOD of 0.072 ng/mL [286]. Both of these sensors are well within the range necessary for oral cancer patient identification through IL-8 biomarkers [285,286].

### 4.3. MNPs in Novel Coronavirus Sensing

The 2019 novel coronavirus (2019-nCoV) is responsible for the COVID-19 pandemic of the year 2020 [292]. It is also known as severe acute respiratory syndrome coronavirus 2 [8]. It is a ribonucleic virus, i.e., an RNA virus that is known to infect and attack various parts of the body, but causes most damage to the respiratory and cardiovascular system [292,293]. Besides economic impact, the long-term lockdown due to COVID-19 will have a severe mental health impact on both individuals and society as a whole [294]. One of the proven and most effective way to control the spread and minimize the 2019-nCoV impact is through testing. Asymptomatic and pre-asymptomatic individuals infected with 2019-nCoV are also highly contagious [292]. As a result, researchers around the globe have invested their time and knowledge in developing POCT systems that can be used for rapid, accurate, and early detection of the virus [8,292].

**Figure 22:**
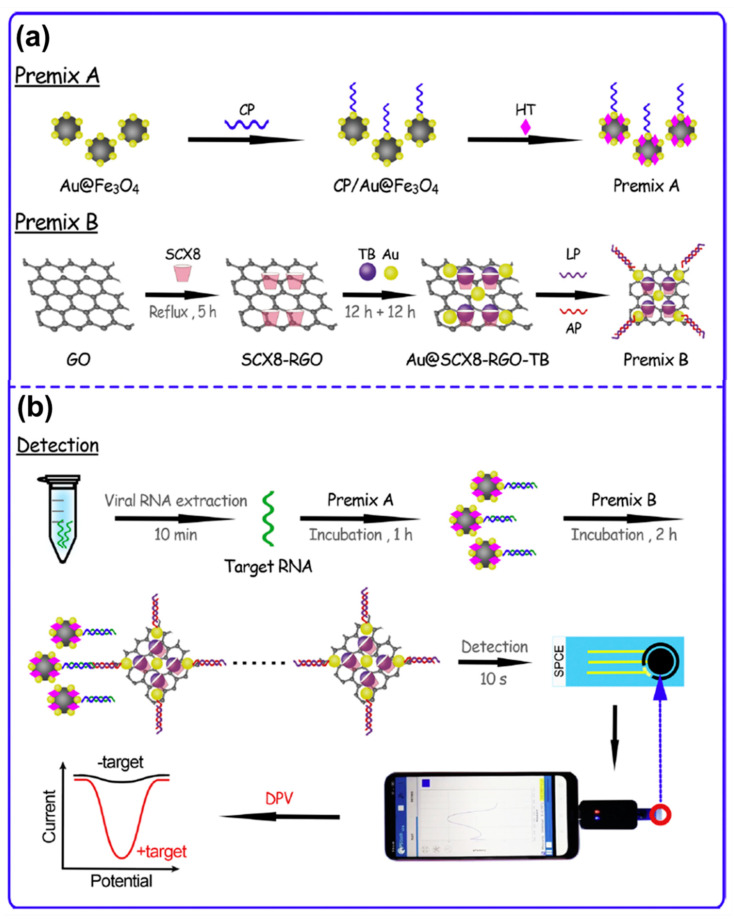
Schematics for the fabrication of a plug-and-play super-sandwich electrochemical immunosensor for 2019-nCoV. (**a**) shows the synthesis of Premix A and B. (**b**) combining Premix A with 2019-nCoV RNA and preparation of the super-sandwich for the detection 2019-nCoV through a smartphone [296]. Reprinted with permission from [296], Copyright © 2020 published by Elsevier B.V.

ECBs can be used to reach all these goals in a cost-effective manner [293]. There are various proposals for the synthesis of ECB strips that can effectively detect 2019-nCoV [294,295]. Smartphone-based ECBs for ultrasensitive detection of 2019-nCoV were reported by Zhao et al [296]. The reported aptasensor utilized a super-sandwich setup for the detection of 2019-nCoV through a “signal on” process with the DPV technique. The sensor fabrication process and its 2019-nCoV detection process is shown in Figure 22a. For the sandwich setup, initially, thiolated capture probes (CPs) were immobilized on the surface of the Au@Fe_3_O_4_ NPs (Premix A). Calixarene8 (CX8) was used for enhancing the electrochemical activity of TB through a supramolecular interaction process. Essentially, the host–guest complex utilized AuNPs, CX8, TB, LP (label probe) and an rGO system. Finally, the immobilized host–guest system was combined with the AP (auxiliary probe) to make the final modifications (Premix B). After extraction, 2019-nCoV RNA was first incubated with Premix A for 1 h, followed by 2 h incubation with Premix B. The sensor showed a significant increase in DPV current signal for the 2019-nCoV RNA combined Premix A and B setup compared to just the Premix B setup. The reported sensor was also tested on 2019-nCoV active and recovering patients. The proposed sensor showed higher effectiveness compared to the reverse transcription real-time polymerase chain reaction (RT-qPCR) for 2019-nCoV detection from both active and recovering patients. The sensor can be used with a smartphone, in a plug-and-play system for the effective POCT diagnosis of 2019-nCoV [296]. Once commercialized, these portable ECBs could be highly effective in contact tracing and controlling the spread of 2019-nCoV.

## 5. Advances in POCT Devices: Prospects and Challenges in the Clinical Transition of ECBs

**Figure 23:**
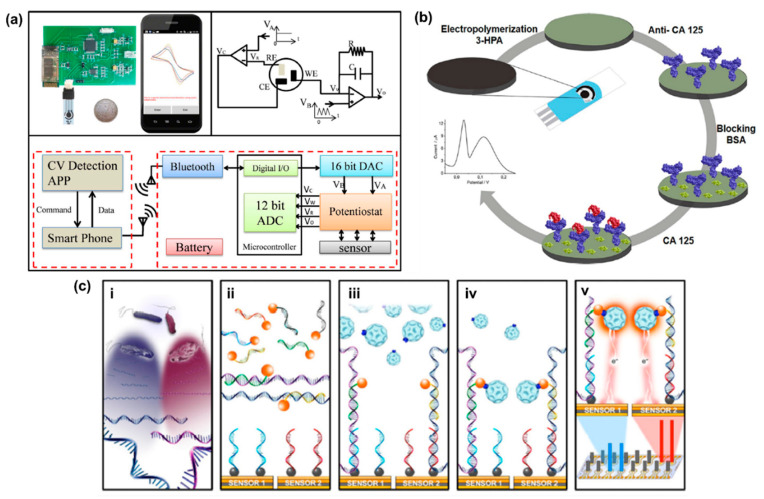
Schematics of hand-held ECBs with potential for POCT. (**a**) a smartphone-based ECB for the sensitive detection of glucose from blood samples. The figure shows the circuit setup that was used for connecting the ECB with the smartphone and detecting glucose through the CV technique [298]. (**b**) fabrication of a label-free immunosensor on a screen-printed carbon electrode (SPCE) strip for the detection of CA125 biomarkers from serum samples through the DPV technique [71]. (**c**) mechanism proposed for a clinically tested ECB for the simultaneous detection of multiplex pathogens for the diagnosis of urinary tract infection; (i) lysis of different bacteria through identification of 16S rRNA; (ii) hybridization with detector probes; (iii) combining with the capture probe immobilized on the electrode surface; (iv) binding of anti-fluorescein HRP tag to form sandwich system; and (v) generation of i-t current signal for a fixed potential that corresponded to the concentration of different bacteria present in the system [300]. Reprinted with permission from [71,298], Copyright © 2020 and 2020 published by Elsevier B.V. [300], Copyright © 2020 and 2020 published by the American Urological Association. DAC: Digital-to-analog converter.

POCT provides health experts and patients with the opportunity to monitor health conditions and diagnose a disease quickly and accurately. Furthermore, the introduction of personalized heath care would be possible with the large-scale implementation of POCT [6,8,12]. This would allow for early-stage detection of cancer biomarkers, senescent β-cells in type-I diabetes patients, or beta-amyloids in Alzheimer’s disease patients [65,297,298]. Because of this, research in ECBs has focused on developing prototype biosensor devices that can be used with mobile or other convenient electronic systems for the rapid analysis of biomolecules [6,65]. Wearable paper ECBs were reported for the detection of sulfur mustard that used EmStat^3,^ a portable potentiostat [12]. A glucose sensor was developed that could be used with a smartphone for blood glucose level detection [298]. Castro et al. reported the fabrication of label-free immunosensor strips for the sensitive detection of CA125 cancer biomarkers from human serum sample [71]. An ECB device has been reported for the rapid detection of the COVID-19 virus, that could be used as an alternative to the RT-qPCR-based 2019-nCoV test [296]. Figure 23 shows the fabrication and detection mechanism of these sensors.

ECBs fall within of one of the five EATs that have been discussed in the Section 2.1. These techniques utilize Ohm’s law, the Nernst equation, and other well-established theories of electrochemistry [82]. For instance, the commercial glucose sensors utilize a biocatalytic process for blood glucose detection through voltammetry or amperometry techniques. There are ECBs that have been developed to communicate with smartphones through micro-USB ports, audio channels, or even wirelessly [82]. Despite the increasing research and steady development in POCT electrochemical devices, there are almost no ECBs in the market for consumer use other than the glucose sensor for diabetes patients [48]. These devices are often used only in laboratory testing and do not progress towards clinical trials. Long trial times for evaluation and lack of funding are some of the reasons for such shortcomings. ECBs utilize enzymes, antibodies, proteins, peptides, and many other biomolecules as BRCs. All of these have their advantages and limitations. The storage stability, data reproducibility, and complicated sensor fabrication process are some of the key issues that has hindered the clinical transition of ECBs [49,50,51]. Discussion of Table 1 on the MNP-based ECBs shows that despite the use of diverse biomolecules, there still lacks the development of size- and shape-controlled MNPs; there is very little use of affibody molecules. There are more than 353,000 research studies listed on the clinicaltrials.gov website. However, when we searched for the term “electrochemical biosensor”, only three studies were found. Of these, two were ongoing and one study had been completed. The clinical trial identification number for the completed study is NCT00591240, and they published two reports based on their findings [299,300]. The completed study was conducted with the help of 116 patients that utilized ECBs for the detection of pathogens that cause urinary infections [300]. The sandwich mechanism was employed by the sensor for simultaneous detection of multiple bacterial species (*E. coli*, *Enterococcus*, etc.). Figure 23c shows the fabrication process and depth effect in the ECB. The researchers compared their ECB with other urine cultures and concluded that the ECB design needed further modification to improve the LOD and specificity [300]. 

There are reports of ECBs for detecting bacterial systems that showed better performance due to the incorporation of MNPCs for anchoring the biorecognition component [31,301]. In one of the ongoing studies (NCT04053140), researchers were using microneedle array-ECBs for administrating Benzylpenicillin IV 1200 mg. The work was in Phase 1 trial, and had not published any results at time of writing. The other clinical trial was studying the ECB system towards detection of leukocyte esterase biomarkers for periprosthetic joint infection (PJI) (NCT04390607). The studies were aiming to test the ECB on subjects that underwent revision joint surgery. The study was set to begin from November 2020. From Table 2 and Table 3, it becomes clear that using MNPs can significantly enhance the performance, stability, and reproducibility of ECBs, and lower the production cost at the same time. Hence, thorough investigation of the impact of MNP properties on the design and effectiveness of ECBs might help to realize the POCT in the near future [297,302]. The following key points might be inferred based on our analysis of more than 250 articles on ECBs that utilized MNPs for improving their practical applicability:

- ECBs that utilize MNPs or MNPCs usually show higher sensitivity, stability, and wider linear range compared to those that do not utilize MNPs. However, it is important to choose which MNPs are most compatible with a specific BRC. Therefore, research into MNP and BRC compatibility might greatly enhance the stability of the fabricated ECBs.
- Using bi- or tri-metallic NPs significantly enhances the performance of ECBs. The possible reason for this is that the metal–metal interaction helps in lowering the HOMO-LUMO energy gap. This in turn allows for more active sites on the MNPs. Hence, using multimetallic NPs that interact synergistically with each other will allow for stronger interactions with the BRCs.
- MNPs with cubic, pyramidal, oval, and other unique shapes show higher catalytic activity and have an increased surface area compared to the commonly employed spherical MNPs. This would allow for immobilization of a greater amount of BRCs. At the same time, the edge sites of these MNPs show higher activity compared to the basal sites.
- MNPs that have QD or core shell-like structure show some unique physical, chemical, and electronic properties. These unique properties usually make them highly desirable for fabrication of ECBs.
- Green synthesis of MNPs is becoming ever popular. This not only allows for the preparation of MNPs in an environmentally friendly way, but also introduces various functional groups on the MNP surface. These functional groups, when properly utilized, might help in the robust anchoring of BRCs and enhance the stability and overall activity of the ECBs.
- MNPs interact differently with various CNMs. Hence, it is essential to properly choose the MNPs and CNMs before composites can be prepared for fabricating effective ECBs. Future research should focus on understanding the fundamental properties of various MNPCs. This would allow for the intelligent designing of MNPCs for fabricating ECBs.
- The screen-printing technique is most commonly used in the fabrication of ECB strips. However, methods such as inkjet printing, doctor blading, and aerosol-assisted chemical vapor deposition should be explored for determining the best approach for the fabrication of ECB strips.
- Aside from the above-mentioned topics, ECB researchers should work towards the commercialization of their laboratory models. This would then reveal the limitations of their proposed systems, and make way for future research that would help to overcome these shortcomings.

The authors hope that this review will help researchers to see the importance of the proper utilization of the various properties of MNPs in developing effective ECBs. Furthermore, this would allow the fabrication of cost-effective ECBs with high stability and accuracy for the POCT of small biomolecules, cancer biomarkers, and other pathogenic diseases.

## Figures and Tables

**Table 1:** The table describes ECBs that utilized MNPs with various sizes and shapes along with various conducting nanomaterials (CNMs) for biomolecule sensing. The biorecognition components, biointeraction process, and EATs used in these ECBs are also mentioned.

Transducer	Biorecognition Component	MNP	MNP Size	MNP Shape	Biointeraction and EAT	Analyte	References
Ab-N,S-GQDs@AuNP–PANI	anti-HEV antibody	AuNP	6–14 nm	spherical	Bioaffinity immunosensor, pulse impedance	HEV	[4]
AuNP/SWCNTs/PDA/gold electrode	probe DNA	AuNP	15 nm	oval	Bioaffinity aptasensor, LSV	target DNA	[23]
HRP@PGA-C/AgNP	HRP	AgNP	5–8 nm	spherical	Enzyme-based Biocatalytic, amperometry	H_2_O_2_	[26]
PtNPs/MWCNT/PEDOT	glutamate oxidase	PtNP	~12–20 nm	spherical	Enzyme-based Biocatalytic, amperometry	Glutamate	[27]
PtNP/GR/SPCE	ortho-phenylenediamine	PtNP	400 nm	urchin	Non-enzymatic Biocatalytic, CV	Cotinine	[28]
AuNPs–CS/GR/CPE	single-stranded DNA Aptamer	AuNP	10–20 nm	spherical	Bioaffinity aptasensor, DPV	Activated protein C	[29]
Au/AuNP–avidin-HRP	avidin-HRP	AuNP	40 nm	nanowall	Enzyme-based Biocatalytic, amperometry	DNA Methylation	[39]
MWCNT–AuNPs/GCE	E-cadherin antibody–QD	AuNP	5 nm	spherical	Bioaffinity immunosensor, DPV	Epithelial-mesenchymal transition	[41]
Cu–nanoflower@AuNPs–GO NFs coated Au chip	GOx–HRP–Cu nanoflower	AuNP	20 nm	spherical	Enzyme-based Biocatalytic, amperometry	Glucose	[44]
PB–CS–AuNPs/SPCE	HRP-labeled histamine antibody	AuNP	~50 nm	spherical	Bioaffinity immunosensor, amperometry	HA	[105]
DMF–EC/AuNPs/HRP	HRP	AuNP	~20–30 nm	spherical	Enzyme-based Biocatalytic, amperometry	H_2_O_2_	[62]
BSA/anti-A(1–42)/AuNPs/MPA/Au	monoclonal antibody mAb DE2B4	AuNP	~30 nm	spherical	Bioaffinity immunosensor, SWV	amyloid beta 1–42	[70]
ds-ATPA/TBA on AuNPs–MoS2/GCE	ds-ATPA and TBA	AuNP	10 nm	spherical	Bioaffinity aptasensor, SWV	ATP and Thrombin	[85]
AgNPs@GQDs/CS/GCE	GOx	AgNP	40 nm	spherical	Enzyme-based Biocatalytic, CV and amperometry	Glucose	[155]
GOx/PtNP@SnS2/Nafion/GCE	GOx	PtNP	~20–40 nm	spherical	Enzyme-based Biocatalytic, amperometry	Glucose	[168]
XO/AuNP/PtNP/MWCNT/GCPE	Xanthine oxidase	AuNP PtNP	50 nm 5 nm	Spherical spherical	Enzyme-based Biocatalytic, CV	xanthine	[169]
AgNPs–Aβ/PrP_95–110_/GE	peptide	AgNP	~15 nm	spherical	Bioaffinity aptasensor, LSV	beta-amyloid	[170]

HEV: Hepatitis E virus; GQD: graphene quantum dot; PANI: polyaniline; PDA: poly dopamine; PEDOT: Poly(3,4-ethylenedioxythiophene); SPCE: screen-printed carbon electrode; CS: chitosan; DMF: dimethylformamide; PB: prussian blue; BSA: bovine serum; MPA: 3-mercaptopropionic acid; ATPA: ATP aptamer; XO: Xanthine oxidase.

**Table 2:** A brief description of various ECBs that have been reported from detection of glucose, dopamine (DA), H_2_O_2_, and uric acid (UA).

Transducer	MNP	Biorecognition Component	Analyte	Linear Range	LOD	References
HRP@PGA-C/AgNP	AgNPs	HRP	H_2_O_2_	1–3000 µM	0.35 µM	[26]
Cu–nanoflower@AuNPs–GO NFs coated Au chip	AuNPs	GOx–HRP–Cu nanoflower	Glucose	0.001–0.1 mM	0.018 µM	[44]
DMF-EC/AuNPs/HRP	AuNPs	HRP	H_2_O_2_	25–100 µM	9.1 µM	[62]
AgNPs@GQDs/CS/GCE	AgNPs	GOx	Glucose	0.1–10 mM	0.01 mM	[155]
GOx/PtNP@SnS2/Nafion/GCE	PtNPs	GOx	Glucose	0.1–1 mM 1–12 mM	2.5 µM	[168]
GOx–NiNP/Ni/Au	NiNPs	GOx	Glucose	1–12 nM	0.42 mM	[179]
Ag-doped PANInanocomposites/GCE	AgNPs	AgNP	DA H_2_O_2_	10–90 µM 10–90 µM	0.12 µM 0.03 µM	[183]
Ag/MoS_2_/ITO	AgNPs	Ag/MoS_2_	DA	0.2–50 µM	0.2 µM	[186]
GOx/PtNP/acetic acid-treated LIG	PtNPs	GOx	glucose	0.3 µM–2.1 mM	0.3 µM	[187]
GOx/PVA-Fe_3_O_4_/Sn	Fe_3_O_4_	GOx	glucose	1–30 mM	0.5 mM	[188]
UOx/EDC:NHS/CZTS/ITO	Cu_2_ZnSnS_4_	UOx	UA	50–700 μM	1.3 µM	[184]
UOx/Au-rGO/ITO	AuNPs	UOx	UA	50–800 μM	7.32 µM	[185]
AuNFs/Fe_3_O_4_@ZIF-8-MoS_2_	AuNPs	Fe_3_O_4_@ZIF-8	H_2_O_2_	5 µM–120 mM	0.9 µM	[45]
GCE/HUA/HNT/FAD	HNT	HUA and FAD	H_2_O_2_	1–250 μM	0.49 µM	[189]

PGA: poly(glutamic acid); NF: nanoflower; GOx: glucose oxidase; UOx: uricase; HUA: humic acid; HNT: Halloysite nanotube; FAD: flavin adenosine dinucleotide; LIG: laser-induced graphene; ITO: indium tin oxide.

**Table 3:** MNP-based ECBs for the detection of cancer biomarkers. The table discusses the transducer design and biomarker detection processes, (*) is used to tag base electrode (transducer).

Nanostructure Biorecognition Molecules Modified Transducer (Base Electrode *)	Type of ECBs	EAT	Linear Range (ng/mL)	LOD (ng/mL)	Reference
Carcinoembryonic antigen (CEA): Colorectal, pancreatic, breast, and liver cancers					
Au*/COOH–rGO/ PdAuPt/antiCEA	Immunosensor	DPV	0.012–85	0.008	[195]
AuNPs/TiO_2_–GR/HRP–Ab_2_ and GCE*/AuNPs/Ab_1_/CEA	HRP-labeled Sandwich immunosensor	DPV	0.005–200	3.33 × 10^−6^	[196]
Fc–AuNPs–Ab_2_ and Au*/LPA–NHS/Ab_1_/CEA	Fc-labeled Sandwich immunosensor	SWV	0.05–20	0.01	[197]
Ag@CeO_2_–Au–Ab_2_ and GCE*/AuNPs/Ab_1_/CEA	Sandwich immunosensor	CV, EIS	0.0001–5	32 × 10^−6^	[198]
MoS_2_/g-C_3_N_4_/PtCu/Ab_2_ and GCE*/AuNPs/Ab_1_/CEA	Sandwich immunosensor	i-t	0.0001–80	3 × 10^−5^	[199]
Fe_3_O_4_@AuNPs–DNA(S1)–S2–S3–CEA-Exoll/Hemin	Magnetic aptasensor	DPV	0.1–200	0.0004	[200]
Ag–PANI@MnO_2_/Ab_2_ and GCE*/AuNPs/Ab_1_/BSA/CEA	Sandwich immunosensor	DPV	0.0005–80	0.00017	[201]
Cu–MOFs–TB/PDA/Ab_2_ and GCE*/MWCNT/CS/Ab_1_/CEA	TB-labeled sandwich immunosensor	DPV	2 × 10^−5^–200	3 × 10^−6^	[202]
Mag–SPCE*/AuNP–MnO_2_/ Fe_3_O_4_@Au/antiCEA	Immunosensor	LSV EIS	0.001–100	0.0001 (LSV) 0.0003 (EIS)	[203]
GCE*/PDA–rGO/Ag–Au/antiCEA	Immunosensor	CV	0.001–80	2.86 × 10^−4^	[204]
GCE*/HNF/AuNP/cMWCNT/antiCEA	immunosensor	EIS	0.4–125	0.09	[205]
CSH/Ab_2_/BSA and GCE*/MoS_2_–Au/Ab_1_/CEA	Sandwich immunosensor	DPV	0.0001–80	3 × 10^−5^	[206]
GCE*/Au@PDA@Fe–MOF/NH_2_–aptamer/BSA/CEA	Aptasensor	DPV	1 × 10^−6^–1000	3.3 × 10^−7^	[207]
GCE*/CNT@PAMAM/CdSe NP/Ab_2_/CEA/Ab_1_/Fe_3_O_4_	Cation-labeled sandwich immunosensor	SWV	0.005–50	0.0017	[208]
MWCNTs/CoS_2_@PANI/HRP and GCE*/Au/Ab_1_/BSA/CEA	HRP-labeled sandwich immunosensor	i-t	0.001–40	0.0003	[209]
GCE*/NCMT@Fe_3_O_4_@CuSiO_3_/ConA/CEA/AuNC-aptamer	Cation-labeled aptasensor	DPV	0.03–6	5.38 × 10^−3^	[210]
CeO_2_-MoS_2_/Pb^2+^/Ab_2_ and GCE^*^/Au/Ab_1_/BSA/CEA	Cation-labeled sandwich immunosensor	SWV	0.001–80	0.0003	[211]
MoS_2_@Cu_2_O/Fc/Ab_2_ and GCE*/Au/Ab_1_/BSA/CEA	Fc-labeled sandwich immunosensor	SWV	0.001–80	3 × 10^−5^	[212]
Au*/Ni-Co-PBA/aptamer/CEA	Aptasensor	EIS	0.001–5	7.4 × 10^−7^	[213]
GCE^*^/MWCNT-SO_3_H/Rh@Pd ND/Ab_1_/BSA/CEA	Immunosensor	DPV	2.5 × 10^−5^–100	8.3 × 10^−6^	[214]
GCE^*^/rGO-PtAu NP/antiCEA	Immunosensor	SWV	1 × 10^−5^–100	7 × 10^−6^	[215]
Ag-MOF/AuNP/Ab_2_ and GCE^*^/MWCNT/Ab_1_/BSA/CEA	Ag(I)-labeled sandwich immunosensor	DPV	0.05–120	8 × 10^−6^	[216]
GCE*/GO-AuNP/antiCEA	Immunosensor	SWV	1–40	0.0158	[217]
AgNP@Strp-HRP/Ab_2_ and GCE*/T–GO/AuNP@Strp/Ab_1_/CEA	HRP-labeled sandwich immnusensor	DPV	0.0001–0.005	7.5 × 10^−5^	[218]
**Fc**: ferrocene; **LPA-NHS**: Lipoic acid N-hydroxysuccinimide ester; **rGO**: reduced GO; **HNF**: honey nanofibers; **BSA**: bovine serum; **PAMAM**: poly(amidoamine); **Strp**: streptavidin;					
**Prostate specific antigen (PSA)**: Prostate cancer					
Ab_2_–HRP and GCE*/C_60_/PANI@PdNP/Ab_1_/BSA/PSA	HRP-labeled sandwich immnusensor	CV	0.00016–38	1.95 × 10^−5^	[219]
GCE*/MoS_2_–GA@AuNP/antiPSA/BSA	Immunosensor	DPV	1 × 10^−5^–50	3 × 10^−6^	[220]
Cu_3_(BTC)_2_/Ab_2_ and Au*/PG@PDA/Ab_1_/CAS/PSA	Sandwich immunosensor	i-t	0.1–10	0.025	[221]
d-Ti_3_C_2_T_x_@AuNP/Ab_2_ and GCE*/ATP–GO@AuNP/Ab_1_/BSA/PSA	Sandwich immunosensor	DPV	0.00001–0.001	3 × 10^−6^	[222]
SPCE*/AuNP@aptamer/MCH/PSA	Aptasensor	DPV	0.001–200	7.7 × 10^−5^	[223]
GCE*/CeO_2_–MnO_2_/antiPSA	Immunosensor	SWV	0.005–50	0.005	[224]
SPCE*/GO/antiPSA	Immunosensor	DPV	0.75–100	0.27	[225]
Au*/MPA/f-PSA/BSA andAu*/MPA/t-PSA/BSA	Immunosensor	EIS	0.00002–200	3 × 10^−6^ (f-PSA) 4 × 10^−6^ (t-PSA)	[226]
Ab_2_-CdNi QDs and GCE*/Fe_3_O_4_@TMU-10(MOF)-CS/Ab_1_/BSA	QD-labeled sandwich immunosensor	DPV	0.001–100	0.00045	[227]
Ab_2_-HRP and GCE*/RC_60_/CuNP@HQ/Ab_1_/BSA	HRP-labeled Sandwich immunosensor	DPV	0.005–20	0.002	[228]
GCE^*^/GQDs–CS–Naf-IL/MWCNT–GR–IL/PPY-MoS_2_–il–AuPt NP/aptamer/PSA	Aptasensor	SWV	0.0005–0.35	0.00014	[229]
GCE^*^/MWCNT@AuNP-GQD/Ab_1_/BSA/PSA	Immunosensor	EIS	0.001–10	0.00048	[230]
CPE*/Gr-Fe_3_O_4_ NP/antiPSA	Immunosensor	DPV	1–150	0.38	[231]
Au IDE*/16-MUA+EDC/NHS/antiPSA	Immunosensor	f-EIS and nf-EIS	0.01–100 and 0.5–1000	0.01 and 0.5	[232]
Au*/Peptide/GO@AgNP	ECB	LSV	0.005–20	0.00033	[233]
GCE*/rGO-NH_2_/AgPtPd-COOH/antiPSA	Immunosensor	DPV	4 × 10^−6^–300	4 × 10^−6^	[234]
GCE*/AuNP/rGO@AuNP/antiPSA	Immunosensor	SWV EIS	5.5 × 10^−8^–0.25; 1–36 (SWV) and 0.0018–41.15	0.06; 0.002 (SWV) and 0.006 (EIS)	[235]
GCE*/PANI@AuNP/Pep/aptamerPSA/BSA	Aptasensor	DPV	0.0001–100	8.5 × 10^−5^	[236]
Ab_2_-HRP and CASPAuE-MFD*/Magb-Ab_1_/BSA/PSA	HRP-labeled Sandwich immunosensor	i-t SWV	0.001–10	0.00084 (i-t) 25.4 fM (SWV)	[237]
Ab_2_/AuNP@cys-AgNP@Cu^2+^ and GCE*/GS@SnO_2_/Au@Pt/Ab_1_/BSA/PSA	Cu^2+^-labeled sandwich immunosensor	SWV	0.01–100	0.00384	[238]
GCE*/fMWCNT@AuNP-0.5/antiPSA and GCE*/fMWCNT@AuNP-50/antiPSA	Immunosensor	CV	0–4 and 0–6	85 and 56	[141]
GCE*/AuNP/Ab_1_/BSA/PSA/Ab_2_-S_0_/DNA concatemer(S_1_-S_2_)-AgNP Signal probe–DNA concatemer (S_1_-S_2_)–AgNP	Ag-labeled sandwich immunosensor	DPV	0.0001–75	3.3 × 10^−5^	[239]
Ab_2_–HRP and SPE*/CS/AuNP/Ab_1_/BSA/PSA	HRP-labeled Sandwich immunosensor	SWV	1–18	0.001	[240]
SPCE*/rGO@ thionine–AuNP/DNA aptamer/	Aptasensor	DPV	0.05–200	0.01	[241]
PtNP-Ab_2_/BSA–CuNP and GCE^*^/AuNP/Ab_1_/BSA/PSA	CuNP-labeled sandwich immunosensor	SWV	0.0005–100	14.57 × 10^−5^	[242]
Au@Ag-Cu_2_O/Ab_2_ and GCE*/Au@N–GQDs/Ab_1_/BSA/PSA	Sandwich immunosensor	i-t	1 × 10^−5^–100	3 × 10^−6^	[243]
**CAS**: casein; **MCH**: 6-mercapto-1-hexanol; **QD**: quantum dot; **MPA**: 3-mercaptopropionic acid; **16-MUA**: 16-[Mercaptoundecanoic acid; **EDC**: N-(3, Dimethylaminopropyl)-N-ethyl-carbodiimidehydrochloride; **NHS**: Lipoic acid N-hydroxysuccinimide ester; **CASPE-MFD**: commercially available screen-printed electrode-based microfluidic devices; **AE**: gold electrode;					
**Cancer Antigen 125 (CA125)**: Ovarian cancer, breast cancer, lymphoma					
Ab_2_–Suc–Chi@MNPs–TB and GCE^*^/PAMAM/AuNP–3DrGO–MWCNT/Ab_1_/Glu/CA125	TB-labeled sandwich immunosensor	SWV	0.0005–10 and 10–75 U/mL	0.006 mU/mL	[244]
MB–mAb–HRP–CA125 and Au*/Aptamer	HRP-labeled sandwich aptasensor	CV	2–100 U/mL.	0.08 U/mL	[245]
Ag–PPy–pAb_2_ and ITO*/MB–mAb_1_	Sandwich immunosensor	LSV	0.001– 300 U/mL	7.6 mU/mL	[246]
Ab_2_–GPTMS–SiNPs and Au*/f-GNS/Ab_1_/CA125	Sandwich immunosensor	DPV	1 × 10^−9^–1 × 10^−15^	1 × 10^−15^	[247]
TB or Fc–Probe@Au–TiO_2_@Ab_2_ and Ta*/BDD/VBG–Au/Ab_1_–CA125 or CEA	TB/Fc-labeled sandwich immunosensor	DPV	CA125: 0.5–100 mU/mL CEA: 0.0005-0.1	CA125: 0.09 mU/mL CEA: 0.00015	[248]
SPCE*/rGO/thionine /AuNPs/antiCA125/BSA	POCT immunosensor	DPV	0.1–200 U/mL	0.01 U/mL	[249]
ITO^*^/AgNPs–PAN–oxime NFs/aptamer/cDNA–MB/CA125	MB-labeled aptasensor	DPV	0.01–350 U/mL	0.0042 U/mL	[92]
GCE^*^/AgNPs–GQD/antiCA125/BSA	Immunosensor	DPV SWV	0.01–400 U/mL	0.01 U/mL	[250]
Hollow MB–PDA–Ab_2_ and GCE*/Au–rGO/Ab_1_/BSA/CA125	MB-labeled sandwich immunosensor	DPV	0.0001–100 U/mL	336 nU/mL	[251]
GCE*/ATA–CNT–MSA; CdS–Ab_2_/AFP/Ab_1_; ZnS–Ab_2_/CEA/Ab_1_; HgS–Ab_2_/CA19-9/Ab_1_; PbS–Ab_2_/CA125/Ab_1_	CdS, ZnS, HgS and PbS-labeled ratiometric multiplex sandwich immunosensor	DPASV	AFP and CEA: 0.0004–10; CA19-9 and CA125: 0.004–100 U/mL	AFP: 0.00011; CEA: 0.0023; CA19-9: 0.68 mU/mL; CA125: 1.4 mU/mL	[252]
SPCE*–AuNP/antiCA125/BSA and SPCE*–PtNP/antiCA125/BSA	Immunosensor	EIS	450–2916	AuNP: 419; PtNP: 386	[253]
GCE*/Ag–DPA–GQDs/CysA–AuNP/antiCA125/BSA/CA125	Immunosensor	DPV	0.001–400 U/mL	0.001 U/mL	[254]
SPE*/Au–AgNPs/antiCA125/BSA (ISA) and SPE*/Au–AgNPs/CysA/antiCA125/BSA (ISB)	Immunosensor	EIS	ISA: 1–500 IU/mL; ISB: 1–1000 IU/mL	1.03 IU/mL	[255]
GCE/FA@H–PANI@CS–HCl/Ab–Ag@Co_3_O_4_/BSA/CA125	FA-labeled immunosensor	DPV	0.001–25	0.00025	[256]
Ab_2_–AuNPs–LOx and GCE*/GO/MWCNT/AuNPs–CS/Ab_1_/BSA	Enzymatic immunosensor	i-t	0.01–0.5 and 0.5–100 U/mL	0.002 U/mL	[257]
AuNP–Ab_2_–Cd^2+^ and ITO*/GNR/Ab_1_/CA125	Cd^2+^-labeled sandwich immunosensor	DPV	20–100 U/mL	3.4 U/ mL	[258]
GCE*/p(CTAB-CS)-AuNP/antiCA125/BSA	Immunosensor	DPV	0.001–400 U/mL	0.001 U/mL	[259]
GCE*/PDA/ERGO/CysA–AuNPs/antiCA125–HRP/BSA	HRP-labeled immunosensor	SWV	0.1–400 U/mL	0.1 U/mL	[260]
**TB**: toluidine blue; **MB**: methylene blue; **GNS**: gold Nanostructures; **GPTMS**: glycidyloxypropyl trimethoxysilane; **BDD**: boron-doped diamond; **VBG**: vertical boron doped graphene; **FA**: ferro-cenecarboxylic acid; **pCTAB**: poly cetyl trimethylammoniumbromide; **AE**: gold electrode; **GNR**: gold nanorods;					
**Human epidermal growth factor receptor 2 (HER2)**: Breast cancer					
SPCE*/AuNPs–MPA/NSCeO_2_/NHS–PEG– Maleimide/antiCA125/BSA	Immunosensor	DPV	0.001–0.5 and 0.5–20	0.0349	[261]
CdSe@ZnS QDs–Ab_2_ and SPCE*/c-MagBs/Ab_1_/EA/HER2	Cd^2+^-labeled sandwich immunosensor	DPASV	0.50–50	0.29	[262]
AE*/MnFePBA@AuNP/Aptamer/HER2 and AE*/MnFePBA@AuNP/Aptamer/MCF7	Aptasensor	EIS	HER2: 0.001–1; MCF7: 500-5 × 10^4^ cell/mL	0.000247; 36 cell/mL	[263]
SPCE*/Ab_1_/HER2–ECD/Ab_2_/CdSe@ZnS QDs	Cd^2+^-labeled sandwich immunosensor	DPASV	10–150	2.1	[264]
AE*/CDs@ZrHf–MOF/Aptamer/HER2	Aptasensor	EIS	0.001–10; 1000–1 × 10^5^ cell/mL	HER2: 19 × 10^−6^; MCF7: 23 cell/mL	[265]
Ab_2_–CDI–PbS QDs and SPCE*–COOH/EDC–NHS/Ab_1_	Pb^2+^-labeled sandwich immunosensor	SWV	1–100	0.28	[266]
GCE*/ErGO–SWCNT/AuNP/Aptamer/HER2	Aptasensor	EIS	0.0001–1	5 × 10^−5^	[267]
GNR–Pd SS–Aptamer–HRP and AE*/DNA tetrahedron/BSA/HER2	HRP-labeled sandwich aptasensor	DPV	10–200	0.15	[268]
ITO^*^/MoO_3_@rGO/APTES/antiHER2/BSA	Immunosensor	DPV	0.001–500	0.001	[269]
GSPE*/AuNPs/antiHER2 affibody/MCH/HER2	Affisensor	EIS	0–4 × 10^4^	6000	[100]
Ab_2_–AuNPs–dC_20_ AE*/peptide/MCH/HER2	DNA-labeled sandwich immunosensor	SWV	0.0001–1	0.0005	[270]
Ab_2_/Hyd@AuNPs–APTMS–Fe_3_O_4_ and GCE*/Fe_3_O_4_–APTMS/Ab_1_/BSA/HER2	Sandwich immunosensor	DPV	0.0005–50	2 × 10^−5^	[271]
**PEG**: polyethylene glycol; **MagB**: magnetic bead; **CDI**: carbonyldi-imidazole; **MCH**: 6-mercapto-1-hexanol; **APTES**: 3-aminopropyltriethoxysilane; **APTMS**: 3-aminopropyltrimethoxysilane; **AE**: gold electrode;					
**α-fetoprotein (AFP)**: Gastrointestinal tumor and liver cancer					
GCE*/PGNR/AuNPs/antiAFP/BSA/AFP	Immunosensor	DPV	5–60	1.0	[272]
Thiolated LAPS*/MPTES–AuNPs/Aptamer/AFP	Aptasensor	potentiometric	100–1 × 10^5^	92	[273]
AuNP–DNA_2_–MB and AE*/DNA_1_/MCH/Fc–CP/AFP/RecJ_f_	Ratiometric aptasensor	ACV	1 × 10^−5^–100	269.4 ag/mL	[274]
AE*/AlCu MOF_2.5,2.5_/Aptamer/AFP	Aptasensor	EIS	0.001–0.5	0.00012	[275]
Pd@PtNPs–Ab_2_– thionine and AE*/AuNPs/Ab_1_/BSA/AFP	Thionine-labeled sandwich immunosensor	DPV	0.0001–100	0.000035	[276]
Ab_2_–AgNP–HRP and GCE*/PANI–AgNP/Ab_1_/BSA/AFP	HRP-labeled sandwich immunosensor	i-t	0.01–1 and 1–10	0.0047	[277]
GCE*/ZnFe_2_O_4_–AgNP@rGO/antiAFP/BSA/AFP	Label-free immunosensor	CV	0.001–200	0.00098	[278]
GCE*/Cu_3_Pt NPs/antiAFP/BSA/AFP	Immunosensor	DPV	0.0001–10	0.000033	[279]
MO/CNT–AuNP–Ab_2_ and GCE^*^/VG–AuPt/Ab_1_/BSA/AFP	MO-labeled sandwich immunosensor	DPV	1 × 10^−6^–100	7 × 10^−7^	[280]
MoSe_2_ NSs–NH_2_/Au@Pt DNRs–Ab_2_ and GCE*/GS–NH_2_/AuNPs/Ab_1_/BSA/AFP	Sandwich immunosensor	i-t	1 × 10^−5^–200	3.3 × 10^−6^	[281]
**PGNR**: porous graphene nanoribbon; **MPTES**: 3-mercaptopropyltriethoxysilane; **AE**: gold electrode; **BSA**: bovine serum; **MO**: methyl orange; **DNR**: dendritic nanorods;					
**Interleukin-6 (IL-6)**: Colorectal cancer					
MCH/Apt/AuNPs/pATP/pABA/GCE	Sandwich aptasensor	EIS	0.005–100	0.0016	[282]
MCHApt/AuNPs/PPyNPs/SPGE	Structure switching aptasensor	EIS	0.001–15000	0.00033	[283]
**MCH**: 6-mercapto-1-hexanol; **Apt**: aptamer; **pATP**: *p*-aminothiophenol; **pABA**: *p*-aminobenzoic acid; **PPY**: polypyrrole;					
**Interleukin-8 (IL-8)**: Oral cancer					
BSA/Anti-IL 8/β–Ag_2_MoO_4_/ITO	Label-free immunosensor	DPV	1 × 10^−6^–40	0.09	[284]
DNA-templated CdTe/CdS QDs/MB	Aptasensor	ASV	1 × 10^−6^–0.005	3.36 × 10^−6^	[285]
Anti-IL8/AuNPs–rGO/ITO	Label-free immunosensor	DPV	0.5–4	0.072	[286]
**BSA**: bovine serum; **MB**: methylene blue; **ITO**: indium tin oxide;					

## Abbreviations

ADHD	Attention deficit hyperactivity disorder
AFP	Alpha-fetoprotein
Anb	Antibody
AgNPs	Silver NPs
Anb	Antibody
Ang	Antigen
AP	Auxiliary probe
APTES	3-aminopropyltriethoxysilane
APTMS	3-aminopropyltrimethoxysilane
ATPA	ATP aptamer
ASV	anodic stripping voltammetry
AuNPs	Gold NPs
BDD	Boron-doped diamond
BRC	biorecognition component
BSA	bovine serum albumin
CAS	Casein
CASPE-MFD	Commercially available screen-printed electrode-Based microfluidic devices
CDI	Carbonyldi-imidazole
cDNA	Complimentary DNA
CE	Counter electrode
CEA	Carcinoembryonic antigen
CNMs	Conducting nanomaterials
CNT	Carbon nanotube
conA	*Concanavalin A*
CP	Capture probe
CPE	Carbon paste electrode
CS	Chitosan
CSH	Copper silicate hollow spheres
CV	cyclic voltammetry
CysA	Cysteamine
DA	Dopamine
DMF	Dimethylformamide
DNR	Dendritic nanorods
DPV	differential pulse voltammetry
DPASV	differential pulse anodic stripping voltammetry
3D-SP	3D screen printed
EATs	electroanalytical techniques
ECBs	Electrochemical biosensors
ECD	Extracellular domain
EDC	N-(3,Dimethylaminopropyl)-N-ethyl-Carbodiimidehydrochloride
EIS	electrochemical impedance spectroscopy
ELISA	Enzyme-linked immunosorbent assay
ESR	Electron spin resonance
FA	Ferro-cenecarboxylic acid
FAD	Flavin adenosine dinucleotide
FC	Ferrocene
FLGR	few layer GR
fMWCNT	Functionalized MWCNTs
Glu	Glutaraldehyde
GNR	Gold nanorods
GNS	Gold Nanostructures
GO	GR oxide
GOx	glucose oxidase
GPTMS	Glycidyloxypropyl trimethoxysilane
GQD	Graphene quantum dot
GR	Graphene
GRNs	graphene nanoribbons
GRWs	GR nanowalls
HA	Histamine
HCC	Hepatocellular carcinoma
HepG2	Hepatocellular carcinoma
HER2	Human epidermal growth factor receptor 2
HEV	Hepatitis E virus
HNF	Honey nanofibers
HNT	Halloysite nanotube
HP	Hairpin probe
HRP	horseradish peroxidase
HUA	Humic acid
IL	Interleukin
ISE	ion selective electrode
ITO	Indium tin oxide
LBL	Layer-by-layer
LIG	laser-induced graphene
LOD	limit of detection
LOx	Lactate oxidase
LP	Label probe
LPA-NHS	Lipoic acid N-hydroxysuccinimide ester
LSV	linear sweep voltammetry
16-MUA	16-Mercaptoundecanoic acid
MagB	Magnetic bead
MB	Methylene blue
MCH	6-mercapto-1-hexanol
MLGR	multilayer GR
MNPs	Metal nanoparticles
MNPCs	MNP composites
MO	Methyl orange
MOF	Metal organic framework
MPA	3-mercaptopropionic acid
MPTES	3-mercaptopropyltriethoxysilane
MSA	Mercaptosuccinic acid
MWCNT	Multiwall CNT
2019-nCoV	Novel coronavirus
NCMTs	Nitrogen-doped magnetic carbon microtubes
NF	Nanoflower
OC	Ovarian cancer
OHA	Ochratoxin A
pABA	*p*-aminobenzoic acid
PAMAM	Poly(amidoamine)
PAN-oxime	Amidoxime-modified polyacrylonitrile
PANI	Polyaniline
pATP	*p-*aminothiophenol
PB	Prussian blue
pCTAB	Poly cetyl trimethylammoniumbromide
PDA	Poly DA
PEDOT	Poly(3,4-ethylenedioxythiophene)
PEG	Polyethylene glycol
PGNR	porous graphene nanoribbon
PJI	Periprosthetic joint infection
PLA	Polylactic
POCT	Point-of-care testing
PGA	Poly(glutamic acid)
PPY	Polypyrrole
PS	Prostate cancer
PSA	Prostate specific antigen
PTK-7	Protein tyrosine kinase-7
PtNPs	Platinum NPs
PVA	Polyvinyl alcohol
RE	Reference electrode
rGO	Reduced GO
SBMs	Small biomolecules
SLGR	single layer GR
SPCE	Screen-printed carbon electrode
ssDNA	Single stranded DNA
ssRNA	Single stranded RNA
Strp	Streptavidin
SWCNT	Single wall CNT
SWV	square wave voltammetry
TB	Toluidine blue
TBA	Thrombin binding aptamer
TMV	tobacco mosaic virus
UA	Uric acid
UOx	Uricase
VBG	Vertical boron doped graphene
WE	Working electrode
XO	Xanthine oxidase
